# High-throughput analysis of the satellitome illuminates satellite DNA evolution

**DOI:** 10.1038/srep28333

**Published:** 2016-07-07

**Authors:** Francisco J. Ruiz-Ruano, María Dolores López-León, Josefa Cabrero, Juan Pedro M. Camacho

**Affiliations:** 1 Departamento de Genética, Facultad de Ciencias, Universidad de Granada, Granada, Spain

## Abstract

Satellite DNA (satDNA) is a major component yet the great unknown of eukaryote genomes and clearly underrepresented in genome sequencing projects. Here we show the high-throughput analysis of satellite DNA content in the migratory locust by means of the bioinformatic analysis of Illumina reads with the RepeatExplorer and RepeatMasker programs. This unveiled 62 satDNA families and we propose the term “satellitome” for the whole collection of different satDNA families in a genome. The finding that satDNAs were present in many contigs of the migratory locust draft genome indicates that they show many genomic locations invisible by fluorescent *in situ* hybridization (FISH). The cytological pattern of five satellites showing common descent (belonging to the SF3 superfamily) suggests that non-clustered satDNAs can become into clustered through local amplification at any of the many genomic loci resulting from previous dissemination of short satDNA arrays. The fact that all kinds of satDNA (micro- mini- and satellites) can show the non-clustered and clustered states suggests that all these elements are mostly similar, except for repeat length. Finally, the presence of VNTRs in bacteria, showing similar properties to non-clustered satDNAs in eukaryotes, suggests that this kind of tandem repeats show common properties in all living beings.

Eukaryote genomes are plenty of repetitive elements including transposable elements (TEs), tandem repeats, segmental duplications, ribosomal DNA, multi-copy gene families, pseudogenes, etc. which, collectively, constitute the repeatome^1^. Satellite DNA consists of a single sequence tandemly repeated many times, in contrast to tandemly repeated genes (e.g. ribosomal RNA and histone genes) where the repeating unit consists of several different DNA sequences (i.e. genes and spacers). Satellite DNA has been classified into microsatellites, minisatellites and satellites, with no complete consensus about the precise length limits^2^,^3^. Although satellite DNA has traditionally been considered to be junk DNA, some possible functions have been suggested during last years. One of the most accepted functional roles for satDNA is its implication in centromeric function^4^, but other possible functional roles have also been suggested in relation with heterochromatin formation through the siRNA pathway^5^,^6^.

The name “satellite DNA” is historical since this kind of repetitive DNA was discovered as a small peak in the CsCl ultracentrifugation profile^7^. Today this technique is not performed to search for satDNA, since it was replaced by other techniques such as DNA renaturation kinetics^8^, restriction digestion and electrophoresis yielding a ladder pattern^9^ and, most recently, by the bioinformatic analysis of a huge collection of short DNA sequences yielded by Next Generation Sequencing (NGS)^10^. Anyway, the term “satellite DNA” is still useful because it is simple, descriptive and profusely used in the literature. On this basis, we are proposing here the name “satellitome” for the whole collection of satDNAs in a genome.

The recent publication of a draft genome of the migratory locust (*Locusta migratoria*) represents a milestone as it is the largest animal genome hitherto sequenced^11^. There is no doubt that it has provided excellent information for performing genomic work in other insects even though annotation is not complete. However, as in other sequenced genomes, information about the repetitive components of the genome is rather scarce, especially for satDNA. We have recently reported microsatellite content in *L. migratoria* at both genomic and cytogenetic levels^12^, but the search for satDNAs through the classical restriction endonuclease digestion and electrophoresis approach failed in this species (MD López-León and P Lorite, personal communication). Up to now, only 21 satDNAs have been reported in 12 orthopteran species, most of them grasshoppers (Supplementary Table S1).

Recently, the use of NGS and new bioinformatic tools like RepeatExplorer^10^ has allowed the high-throughput detection of repetitive DNA, including satellite DNA, the most extreme case being the plant *Luzula elegans* with 37 satDNA families, 20 of which were analyzed by FISH^13^. Here we perform the high-throughput analysis of the satellitome from the information contained in Illumina reads obtained from two individuals of the migratory locust representing the Southern (SL) and Northern (NL) lineages (see methods). By means of stepwise clustering of repetitive DNA^10^ intermingled by substraction of the repetitive elements found in previous steps, we found that the satellitome of *L. migratoria* consists of, at least, 62 different satDNAs with monomer size ranging between 5 and 400 bp. This procedure allowed detection of many poorly abundant satDNAs which would have gone unnoticed through conventional methods. The physical mapping of 59 of them by FISH showed three types of chromosome distribution, with clear predominance of chromosome-specific satDNAs. Finally, this broad catalog of different satDNA families allowed an analysis for general features which provided new insights on the origin and evolution of this part of the repeatome.

## Results

### High-troughput search for satDNAs

In the first run of RepeatExplorer (RE), performed in parallel on Illumina reads from the SL and NL individuals, we found 26 and 21 satDNAs, respectively. We then selected all available reads of each lineage which, after DeconSeq filtering, lacked homology with all satDNAs and other RE clustered sequences previously found in both lineages. A new RE run detected 11 new satDNAs in SL and 9 in NL. After a new step of filtering and RE analysis, we found 2 new satDNAs in SL and 5 in NL. However, the next filtering and RE step failed to show any new satDNA in both lineages, for which reason we stopped this iterative process. At the end, we found 39 satDNAs in the SL individual and 35 in the NL individual. As a whole, these analyses revealed the existence of 62 different satDNA families, 27 of which were assembled in SL, 23 in NL and 12 in both lineages. Subsequent analyses with RepeatMasker to score satDNA abundance and divergence, revealed that 59 out of the 62 satDNAs were present in both lineages, whereas two of them (LmiSat31-8 and LmiSat43–231) were not found in the NL individual, and one (LmiSat62-23) was not found in the SL individual (Table 1). The analysis of variation within these 62 satDNA families showed the presence of 107 sequence variants (i.e. 1–7 per family) (Table 1, Supplementary Table S2, Supplementary Fig. S1). Collectively, all 62 satDNAs represent about 2.39% of the Southern genome and 2.74% of the Northern one (Table 1 and Supplementary Fig. S2). This low amount of satDNA is consistent with the low amount of constitutive heterochromatin revealed by C-banding in this species^14^.

The high number of different satDNA families found in the genome of the migratory locust and the plant *L. elegans*^13^ indicates that eukaryote genomes usually contain a high diversity of satDNA families. During next years, huge amounts of new satDNAs are expected to be uncovered using NGS approaches. We therefore suggest the following simple nomenclature rules to help managing this new information: satDNA name should begin with species abbreviation in Repbase (e.g. Lmi for *Locusta migratoria*) followed by the term “Sat”, a catalog number in order of decreasing abundance (according to the first genome analyzed), followed by consensus monomer length. For instance, the most abundant satDNA in the Spanish genome of *L. migratoria* would read LmiSat01–193. The catalog number would allow differentiating two satDNAs coinciding in length. If, in the future, additional satDNA families were found in other populations of the same species, they should be numbered subsequently to the last one described in previous work. Optionally, if a function is assigned to a satDNA, a reference to it could be added at the end of the name. For instance, since we know that LmiSat07-5 in *L. migratoria* is the telomeric DNA repeat^15^, we could name it LmiSat07-5-tel.

SatDNA abundance was very similar in both genomes, but divergence showed a tendency to be higher in the Northern genome (Supplementary Results S1). To test the reliability of the satDNAs found, we designed primers in opposite orientation for all of them and PCR amplified 59 of them on genomic DNA from Spanish specimens, belonging to the Southern lineage, collected at Cádiz. The three exceptions (LmiSat46–353, LmiSat52–143 and LmiSat57–230) were rare satDNAs which had been found by RepeatExplorer only in the NL genome, whereas RepeatMasker detected them also in the SL individual from the Padul population. However, PCR failed to amplify them in four different SL individuals from the Cádiz population, suggesting population differences for the presence of these rare satDNAs. In addition, LmiSat07-5-tel corresponded with the telomeric DNA repeat (TTAGG)^15^, and was excluded from subsequent analyses because of its known function. Therefore, we will work here with the remaining 58 satDNAs.

The 58 satDNAs showed high variation for monomer length (8–400 bp) and A + T content (29.4–67.6%) (Table 1). Monomer length showed a bimodal distribution, with a 37 bp gap (between 90 and 127 bp) dividing the 58 satDNAs into two groups, one including 26 short satDNAs (8–90 bp) and the other comprising 32 long satDNAs (127–400 bp). The 37 bp gap in monomer length appears to be an oddity of the *L. migratoria* genome, as we have not found such a long gap in *L. elegans* or other grasshopper species (Ruiz-Ruano *et al*., unpublished). Long satDNAs showed higher A + T content and lower divergence than short ones, and the latter show a very high tendency to arise from G + C-rich genomic regions (Supplementary Results S2).

### Short and long satDNAs show similar patterns of chromosomal location

**Figure 1:**
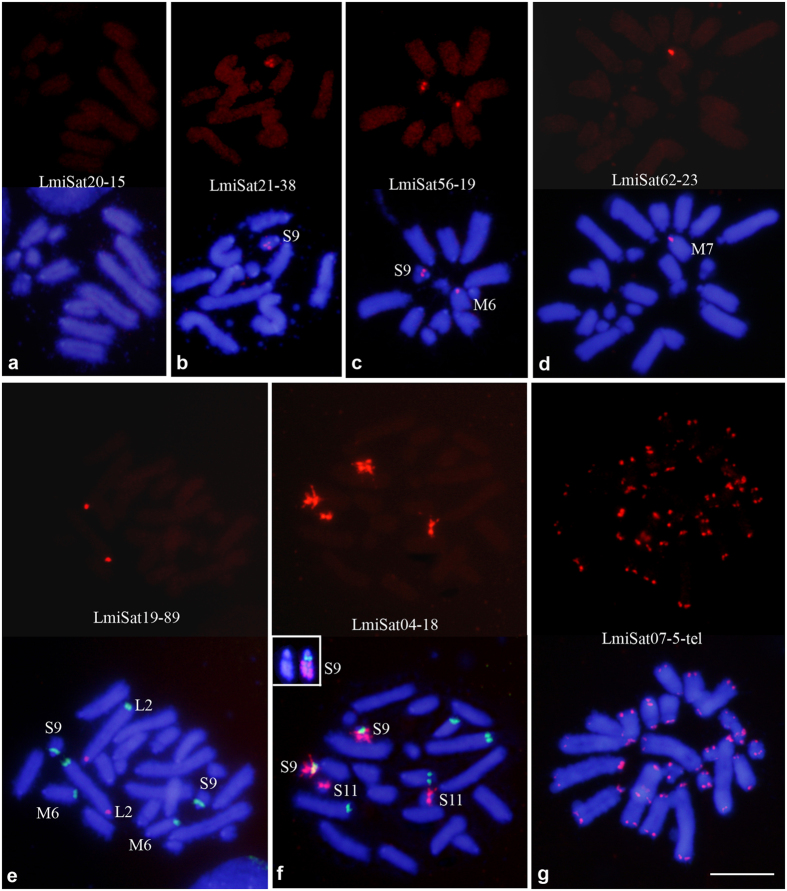
Physical mapping of seven of the short satDNAs found in *Locusta migratoria*, showing the three patterns of chromosome distribution observed: non-clustered (**a**), clustered (**d–g**) and mixed (**b**,**c**). (**a–c)** show haploid mitotic metaphase cells from haplo-diploid embryos, whereas (**d**–**g**) show diploid cells from normal embryos. Each cell is shown in red color for satDNA FISH (upper panel) and merged with DAPI (lower panel). In (**e**,**f**) double FISH was performed to distinguish whether the sat-carrying chromosome was L2 instead of L1 (**e**) and whether S9 carried LmiS at04–18 in addition to rDNA (shown in green color) (**f**). Inset in (f) shows the S9 chromosome stained with DAPI, on the left, and submitted to double FISH for LmiSat04–18 (red) and rDNA (green), on the right, which was selected from another cell showing lower chromosome condensation. Note the presence of three about similar sized satDNA blocks located in interstitial and distal regions of the S9 chromosome. In (**g)** note that LmiSat07–5-tel shows the typical pattern of telomeric repeats.

**Figure 2:**
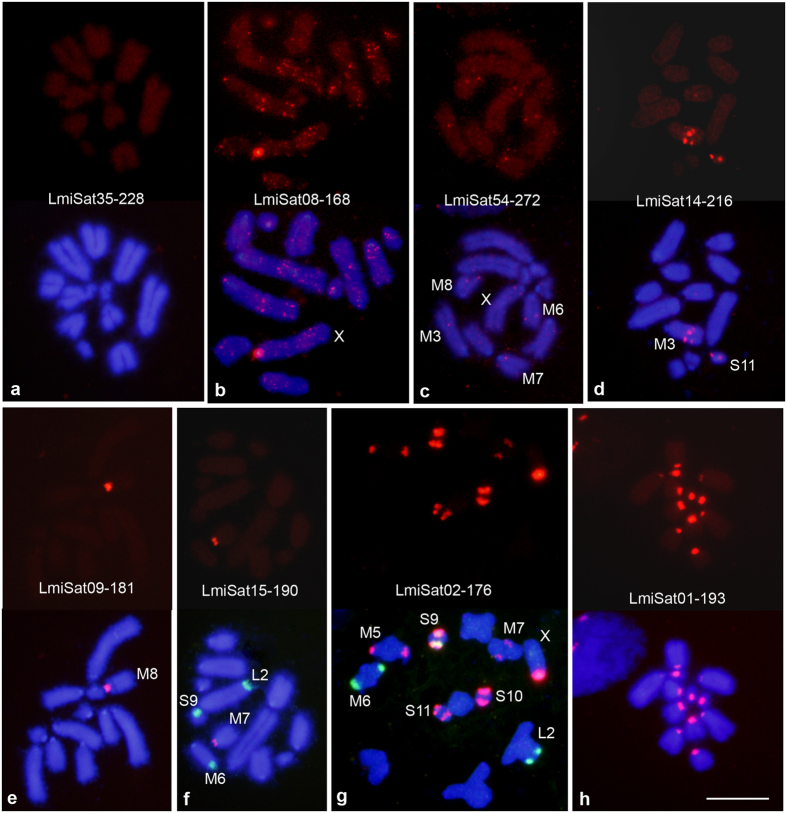
Physical mapping of eight of the long satDNAs found in *Locusta migratoria*, showing the three patterns of chromosome distribution observed: non-clustered (**a**), clustered (**d–h**) and mixed (**b**–**c**). All cells showed here (except that in (**g)**) are mitotic metaphase haploid cells from haplo-diploid embryos obtained in our laboratory. The cell in (**g)** is at meiotic metaphase I and was obtained from an adult male. Each cell is shown in red color for satDNA FISH (upper panel) and merged with DAPI (lower panel). In (**f**,**g)**, double FISH was performed to distinguish whether the sat-carrying chromosome was M6 (harboring rDNA shown in green) or any other medium-sized chromosome. Note in (**h)** the presence of LmiSat01–193 in the pericentromeric regions of all chromosomes.

We performed single FISH analysis for all 58 satDNAs and also double FISH combining a satDNA probe with rDNA or histone gene probes, when needed for accurate identification of the satDNA-carrying chromosomes. Both short (Fig. 1) and long (Fig. 2) satDNAs showed three main patterns at cytological level: clustered at specific chromosome regions (c), non-clustered (nc) and a mixed pattern (m) (Table 1). Depending on satDNA abundance, the non-clustered pattern can go from complete absence of FISH signal to general chromosome brightness above background. The mixed pattern includes both large and very small clusters. The frequencies of c, nc and m patterns did not differ significantly between the two length classes (Supplementary Results S3).

As a whole, the 47 clustered satDNAs (excluding telomeric DNA) showed 89 chromosomal clusters per haploid genome, i.e. 1.89 per satDNA and 7.42 per chromosome pair, on average. Most of them were proximal (52), whereas only 26 were interstitial and 11 distal, with a similar distribution between short and long satDNAs (Supplementary Results S3).

With the exception of the telomeric repeat, short satDNAs were clustered on only 1 or 2 chromosome pairs, whereas clustered long satDNAs were found on 1, 2, 3, 5, 6 or all 12 chromosome pairs, the latter condition being found only for LmiSat01–193, which was located proximal to the centromeric region in all chromosomes, with clusters in the eight shortest chromosome pairs (M4-S11) being larger than those in the four longer chromosomes (L1, L2, X and M3) (Fig. 2g).

**Figure 3:**
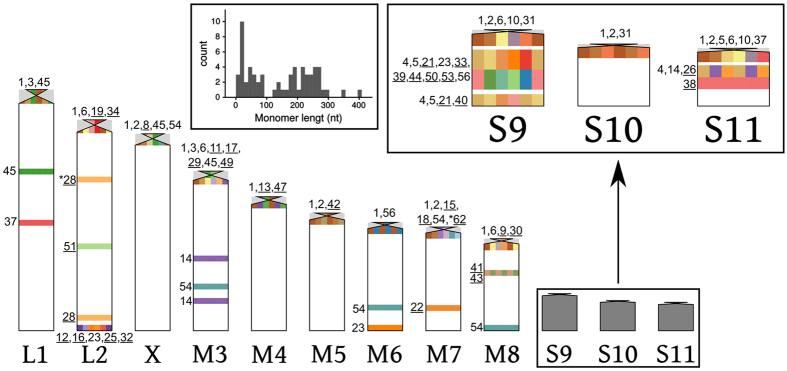
Ideogram showing chromosome location of satDNA clusters mapped by FISH. SatDNAs are noted here only by the catalog number, which is underlined in the case of chromosome-specific families. Polymorphic loci are indicated by an asterisk. Pericentromeric light-grey areas represent constitutive heterochromatin. The inset on the left shows a histogram of monomer lengths for the 62 satDNA families. Note the gap between 90 and 127 bp.

The most frequent pattern, in both short and long satDNAs, was the presence of a large cluster in a single chromosome pair, as was the case for 15 short and 18 long satDNAs (see Supplementary Table S3 and some examples in Figs 1b,d,e and 2e,f), with LmiSat21–38 and LmiSat28–263 showing two clusters in the same chromosome. One satDNA (LmiSat23–223) showed the same location as 45S rDNA in this species. The ideogram in Fig. 3 summarizes the location of all satDNAs.

Excluding the two only satDNAs which were present in all chromosomes, i.e. LmiSat01–193 and LmiSat07-5-tel, the remaining 46 families (19 short and 27 long) of clustered satDNAs (including those showing the mixed pattern) were irregularly distributed among the different chromosomes, with four chromosomes lacking short satDNAs (L1, X, M5 and M8) but all chromosomes carrying one or more different long satDNAs, in addition to LmiSat01–193 (Table 1). Remarkably, the S9 chromosome was the only chromosome carrying more short (10) than long (6) satDNAs.

Only 14 satDNAs (4 short and 10 long) showed clusters in more than one chromosome pair, and this allows testing the equilocality of satDNA distribution. As Supplementary Table S4 shows, short and long satDNAs displayed similar equilocality indices (0.63 and 0.65, respectively) thus reinforcing their similarities in chromosome distribution pattern.

The high number of different satDNAs described here is very useful for chromosome identification in *L. migratoria*, as 15 short and 18 long satDNAs were chromosome-specific markers allowing the direct identification of 9 out of the 12 chromosome pairs, the only exceptions being L1, M6 and S10 (Fig. 3 and Supplementary Table S3). However, these three chromosome pairs can indirectly be identified through their satDNA content pattern, since L1 is the only L-chromosome carrying LmiSat03–195, LmiSat37–238 and LmiSat45–274, M6 is the only M-chromosome carrying LmiSat56-19 and 45S rDNA, and S10 can be identified because it lacks the chromosome-specific satDNAs present in the two similar-sized autosomes (S9 and S11) (e.g. LmiSat04–18, LmiSat05–400 and LmiSat06–185).

A search for the 62 satDNA sequences in the draft genome of *L. migratoria*^11^ revealed that most of them were present in a surprisingly high number of contigs, with very high differences among satDNA families (Table 1), this variation being positively correlated with abundance (Spearman rank correlation: r_s_ = 0.46, N = 58, P = 0.00026). Remarkably, clustered satDNAs showed no significant difference in the number of contigs compared with non-clustered ones (Mann-Whitney test: U = 198, P = 0.23), suggesting that both types of satDNAs are similarly scattered throughout the genome. Therefore, in addition to the large arrays present in the clusters revealed by FISH, clustered satDNAs show many short arrays at many loci across the genome.

### Homologies between satDNAs define five superfamilies

**Figure 4:**
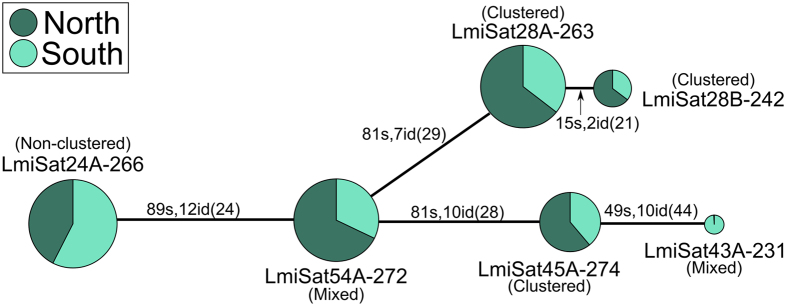
Minimum spanning tree for SF3 superfamily. The link size between haplotypes is proportional to the number of substitutions (s) and indels (id). In brackets, it is indicated the sum of nucleotides involved in the indels. SF3 was composed of six sequences corresponding to five different satDNAs, with lengths ranging between 231 and 274 bp. Note that they constitute a heterogeneous collection of satDNAs showing common descent and displaying all patterns of chromosome location and thus illustrating how long satDNAs may evolve by changing sequence and chromosome location patterns (see Table 1).

A comparison of DNA sequence between the 58 monomer families revealed the existence of similarity between some of them, which allowed defining five superfamilies (Table 1). As shown in Supplementary Fig. S3, superfamily 1 (SF1) includes two long satDNA families: LmiSat01–193, located in pericentromeric regions of all chromosomes, and LmiSat13–259 located only in the M4 chromosome, thus being a case of local derivation of LmiSat13–259 from LmiSat01–193. SF2 includes LmiSat12–273 and LmiSat16–278 both distally located on the L2 chromosome, thus showing satDNA divergence without movement to non-homologous chromosomes. SF3 is composed of five different long satDNA families showing all patterns of chromosome location, thus illustrating how long satDNAs may evolve through sequence diversification and changes in chromosome location patterns (Table 1 and Fig. 4). SF4 includes three long satDNA families (LmiSat26–240, LmiSat37–238 and LmiSat51–241) interstitially located on different chromosomes (S11, L1 and L2, respectively), thus providing evidence for clustering on different non-homologous chromosomes. Finally, SF5 included three short satDNAs (LmiSat31-8, LmiSat50-16 and LmiSat59-16) showing different location patterns, but the reliability of this superfamily is doubtful (see Supplementary Fig. S4 and Supplementary Table S5).

### Homology with other repeated sequences

We found seven satDNA families with homology to sequences from Orthoptera contained in Repbase (Supplementary Table S6 and Supplementary Fig. S5). LmiSat06–185 showed homology with a satDNA previously described in the grasshopper *Caledia captiva*^16^, whereas the six remaining matches in Repbase were with transposable elements (TEs). LmiSat02–176 showed homology with the 5′-end of a Helitron lineage. Two long satDNAs (LmiSat15–190 and LmiSat34–299) showed homology with the CDS of TEs type Gypsy and Polinton, respectively. Likewise, LmiSat29–68 andLmiSat55–90 aligned with a region outside the CDS of two different hAT transposons, and LmiSat19–89 with a DNA transposon described in *L. migratoria*. In addition, LmiSat07–5-tel is the telomeric DNA repeat conserved in the majority of insects^15^. Finally, LmiSat11–37 showed high variation for the number of repeats of a GA microsatellite, for which reason this satDNA showed the highest divergence (56%) and number of variants (7). No other satDNA carrying microsatellites was found. Taken together, these results suggest the possibility that some satDNAs in *L. migratoria* originated from TEs, as in other organisms^17^,^18^.

## Discussion

The 62 satDNA families of *L. migratoria* reported here constitute the highest number of satDNA families ever found in a non-model species. The closest case was the 37 satDNAs reported in the plant *Luzula elegans* within a normal run of RepeatExplorer yielding 291 major repeat clusters with genome proportions of at least 0.01%^13^. Remarkably, the application of our filtering approach to the Illumina reads deposited by Heckman *et al*.^13^ in SRA uncovered 85 satDNA families (grouped into 5 superfamilies), with genome proportions of 0.00035% or higher (Supplementary Table S7). This indicates that our approach improves significantly the bioinformatic analysis for satDNA characterization with RepeatExplorer, by being able to find satDNAs showing 28-fold lower abundance. By performing several successive filtering steps and searches with RepeatExplorer, in each step subtracting those repetitive elements found in previous steps, the chance of finding other poorly represented satDNAs is substantially increased. In *L. migratoria*, the use of genomic reads from two distant populations has also been very useful, allowing detection of satDNAs with abundance as low as 0.00002%. Anyway, it is still conceivable the existence of other less abundant satDNA families which have gone unnoticed with our methodology. Likewise, other individuals from the same or a different population could harbour other satDNA variants or families.

The high-throughput analysis of the satellitome in *L. migratoria* has unveiled several interesting properties of this kind of tandem repeats:

1. The “library” hypothesis^19^ predicts that related species share an ancestral set of different conserved satellite DNA families which may be differentially amplified in each species due to stochastic mechanisms of concerted evolution^20^. The Northern and Southern lineages of *L. migratoria* have shown very similar satellitome catalogs, with only slight differences indicating differential amplification between individuals and/or populations. The intraspecific library shown by the *L. migratoria* satellitome is not composed of completely independent satDNAs, as some of them show similarities enough to constitute five superfamilies. Remarkable conservation was displayed by LmiSat06–185, which showed 72.2% similarity with a satDNA described in *Caledia captiva* (Acridinae subfamily)^16^, a species sharing the most recent common ancestor with *L. migratoria* (Oedipodinae subfamily) about 47 million years ago^21^. SatDNA conservatism has been reported in several organisms, such as beetles genus *Palorus*^22^, the human alpha-satellite DNA (which is highly conserved in chicken and zebrafish)^23^ and satDNAs in some plants^24^, the most extreme case being the persistence of a satDNA for 540 million years in bivalve mollusks^25^. The satellitome opens new avenues to test the library hypothesis at several phylogenetic levels, and library catalogs will be known in unsuspected detail thanks to the NGS techniques.
2. Short and long satDNAs showed the same three patterns of chromosome location (non-clustered, clustered or mixed), and similar equilocal distribution across non-homologous chromosomes. In consistency with previous observations on minisatellites^26^, the short satDNAs observed in *L. migratoria* tend to show high G + C content and sequence divergence, the latter being especially apparent when they are interspersed into euchromatin.
3. The observed equilocality for short and long clustered satDNAs indicates that heterochromatin equilocality^27^ (i.e. the tendency to occupy similar location on non-homologous chromosomes) is actually based on satDNA equilocality, and this pattern may be facilitated by telomere reunion at first meiotic prophase bouquet^28^ which, in the case of acrocentric chromosomes, also implies the reunion of centromeres. Remarkably, short and long satDNAs showed very similar tendency to equilocality.
4. Satellite DNA is frequently located into heterochromatin, and this feature is used to define this kind of DNA. In *L. migratoria*, constitutive heterochromatin is restricted to small pericentromeric regions^14^, which thus include the 52 pericentromeric clusters found for 26 satDNAs. However, the 26 interstitial (for 21 satDNAs) and 11 distal (for 10 satDNAs) clusters are outside constitutive heterochromatin in this species. Therefore, we conclude that satellite DNA is also contained into euchromatic regions, in consistency with recent findings in *Drosophila*^29^ and *Tribolium castaneum*^30^.
5. The high-throughput analysis of the satellitome has been highly informative on satellite DNA evolution. Our present results suggest that previously defined types of satellite DNA^3^ (microsatellites, minisatellites and satellites) show similarities at genomic and cytological levels. We have found here satDNAs with monomer length reaching the domains of typical microsatellites, such as the 5 bp telomeric repeat in *L. migratoria* or several satDNAs in *L. elegans* showing monomer lengths of only 4 or 6 bp (Supplementary Table S7). Likewise, about half of the satDNAs found in *L. migratoria* showed monomer lengths like those defining minisatellites (<100 bp). Remarkably, satDNAs of any length can be clustered or non-clustered at cytological level. Examples of clustered microsatellites can be found in the literature^12^, and our Figs 1 and 2 show that satDNAs between 5 and 400 bp show the same cytological patterns irrespectively of monomer length.
6. The combination of monomer length and number of repeats per locus define array size per locus (ASPL), which actually constitutes the interface between the genomic and cytological levels. Those satDNAs showing ASPL below FISH detection threshold (i.e. about 1.5 kb)^31^, will be non-clustered at cytological level, even though they can be relatively abundant in the genome. Of course, reaching the minimum ASPL to be cytologically observed as a clustered genomic element is more difficult for short satDNAs (especially microsatellites), as many more repeats per locus are necessary (this explains the paucity of clustered microsatellites in the literature). Even long satDNAs can fail to be clustered if ASPL is below 1.5 kbp, but they would become into clustered ones if ASPL would grow above the former threshold at a single genomic locus. For instance, the non-clustered LmiSat24–266 shares the SF3 superfamily with four clustered satellites, two showing the mixed pattern (LmiSat43–231 and LmiSat54–272) and two being clustered (LmiSat28–263 and LmiSat45–274) (see Table 1). A minimum spanning tree of this superfamily suggested a changing dynamics of clustering pattern during evolution (Fig. 4).

**Figure 5:**
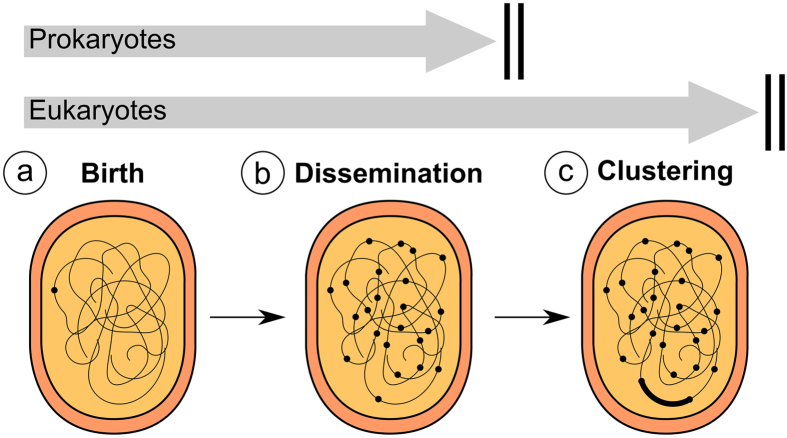
Hypothesis on satellite DNA evolution, based on the fact that all kinds of elements can be found clustered or non-clustered, at cytological level, and show many common satellitome properties. The birth of a satDNA implies a *de novo* duplication of a genomic sequence of two or more bp. This can occurs, for instance, by means of replication slippage in the case of short satDNAs or rolling circle replication in the case of long ones. This gives rise to a short array (<1.5 kb, i.e. the sensitivity FISH threshold) at a single genomic location (small dot in (**a**)). This short array is then disseminated throughout the genome by unknown mechanisms, although transposable elements or rolling circle replication and reinsertion elsewhere might be good candidates (**b**). All satDNAs remain at this stage in prokaryote species, where genomic constraints and natural selection (represented by double vertical bars) pose rigid limits to satDNA accumulation, and some of them remain this way in eukaryotes appearing as non-clustered satDNAs (**b**). In eukaryotes, however, any of the short arrays can undergo local amplification surpassing the 1.5 kb thus becoming into a clustered satDNA and being visible by FISH (**c**). The fact that all clustered satDNAs found in the *L. migratoria* genome were found in many different contigs of the assembled genome provides strong support to the hypothesis that dissemination precedes clustering. Local amplification implies rapid increase in array size and could take place, for instance, by unequal crossing over. Based on our simulation of random genomes of the *L. migratoria* size, satDNA arrays of 15 bp or less can appear by chance at many genomic locations (>15) (Supplementary Table S9). For this reason, all microsatellites and the shortest minisatellites can start their life-cycle at stage (**b)**. Of course, further research is necessary to unveil the precise mechanisms involved in reaching each stage, as those included here are only suggestions based on current literature.

Taken together, the former considerations lead us to suggest a model for satellite DNA evolution (Fig. 5). The first proposals about *de novo* formation of tandem repeats included the joint action of mutation and unequal crossing-over^32^, but other mechanisms, such as slippage replication and/or rolling circle amplification, have also been proposed, with most probable implication of the former in the case of short repeats and the latter in the case of long repeats^26^. Evidences are also accumulating about rolling-circle replication implication in the amplification of satDNAs^33^,^34^, as this mechanism could actually disseminate intragenomically a *de novo* duplicated segment through replication and reinsertion at other genomic locations.

After intragenomic dissemination, many small arrays of a given satDNA will be scattered across multiple genomic locations. Our analysis of the *L. migratoria* draft genome has shown that the immense majority of the 62 satDNAs were contained in many different contigs, suggesting that either most satDNA arrays contain a variety of interspersed sequences or, most likely, they show many different genomic locations. This is also valid for the 33 chromosome-specific clustered satDNAs. The local amplification of short arrays pre-existing at different loci would explain the patterns observed in SF3 and SF4 (see Table 1). In *D. melanogaster*, the 1.688 satellite shows long arrays in the heterochromatin of chromosomes 2, 3 and X, but it is also found as short arrays (1–5 repeats) in the euchromatin of the same chromosomes^29^. Likewise, large blocks of the *Responder* satellite are found in the pericentromeric heterochromatin of chromosome 2 in *D. melanogaster*, but small blocks are also present in the euchromatin^35^. Therefore, a same satellite DNA can be present as short arrays at many cytologically invisible genomic locations and also as long arrays at discrete clusters revealed by FISH.

Remarkably, satellite DNA sequences also exist in bacteria, as variable number tandem repeats (VNTRs) have been reported in several species. For instance, *Bacillus anthracis* shows VNTRs with repeat size ranging from 2 to 36 bp and array size from 1 to 23 repeats^36^, *Salmonella enterica* subsp. *enterica* shows VNTRs with monomer size between 6 bp and 189 bp, with array size of 4–15 repeats^37^, and *Streptococcus uberus* shows VNTRs from 12 to 208 bp monomer length and 2–5 repeats per array^38^. The high similarity of repeat size between bacteria and the animal and plant species analyzed here, and the resemblance of the short arrays length and dissemination pattern, suggest that satellite DNA is a common phenomenon to prokaryotes and eukaryotes. The only difference lies in maximum array size, which is much more limited in bacteria. SatDNA clustering appears to be a eukaryotic innovation presumably facilitated by their large genomes, but total amount of satDNA is likely limited by genomic constraints and natural selection, as in prokaryotes. The fact that 48 satDNAs in *L. migratoria* are clustered, and only 11 are non-clustered, might suggest that clustering is a dead end for satDNA evolution. We suggest that the reverse pathway is conceivable through the action of natural selection when satDNA amounts become a burden. Of course satellitome analysis in other species will throw much light on this subject.

Finally, the equilocal distribution of different clustered satDNA families within a same eukaryotic genome needs an explanation. Certainly, the presence of short arrays acting as seeds at many genomic locations may facilitate contagious equilocal satDNA amplification through unequal crossing over during meiotic bouquet, since this kind of recombination requires the presence of at least short arrays of the same satDNA in different non-homologous chromosomes, and previous dissemination provides them. This is an interesting prospect for future research.

## Methods

### Materials

We collected males and females of *Locusta migratoria* at Padul (Granada) and Los Barrios (Cádiz) in the South of the Iberian Peninsula. Individuals from Cádiz were kept alive in the laboratory to obtain embryo offspring. Due to the extremely high frequency of supernumerary (B) chromosomes in Spanish field populations^39^, it is very difficult to find B-lacking individuals. For this reason, we obtained males and females from a pet shop whose laboratory culture lacks B chromosomes. We crossed a B-carrying male from Padul with a B-lacking female from the culture, and the male offspring was analyzed cytologically to choose one B-lacking individual, following protocols in Cabrero *et al*.^39^. We then extracted genomic DNA (gDNA) with the GenElute Mamalian Genomic DNA Miniprep kit (Sigma) and sequenced the gDNA library (insert size = 226 ± 81 bp) in the Illumina HiSeq2000 platform yielding about 6 Gb data of 2 × 101 nt paired-end reads, ~1× coverage for the gDNA [SRA:SRR2911427]. We also used gDNA Illumina reads (2 × 100 nt) stored in SRA from the *L. migratoria* Chinese individual used for the genome assembling performed by Wang *et al*.^11^ [SRA:SRR764583], randomly selecting the same number of reads as for the Spanish gDNA library. To better characterize the Spanish and Chinese genomes used in this study, we assembled their full mitogenome with MITObim^40^ and built a maximum-likelihood tree with PhyML v3^41^ also including the sequences used by Ma *et al*.^42^ [GenBank:JN858148–JN858212, GenBank:NC_011114-NC011115] to define the Northern and Southern lineages in this species. As Supplementary Fig. S6 shows, the Spanish genome was grouped with the populations from the Southern lineage, and the Chinese genome was included within the Northern lineage.

To test the performace of our satDNA mining protocol in comparison with a typical Repeat Explorer run, we applied it to a gDNA Illumina library (2 × 101 nt) from *Luzula elegans* [SRA:ERR149838] previously analyzed^13^.

### Bioinformatic analysis

**Figure 6:**
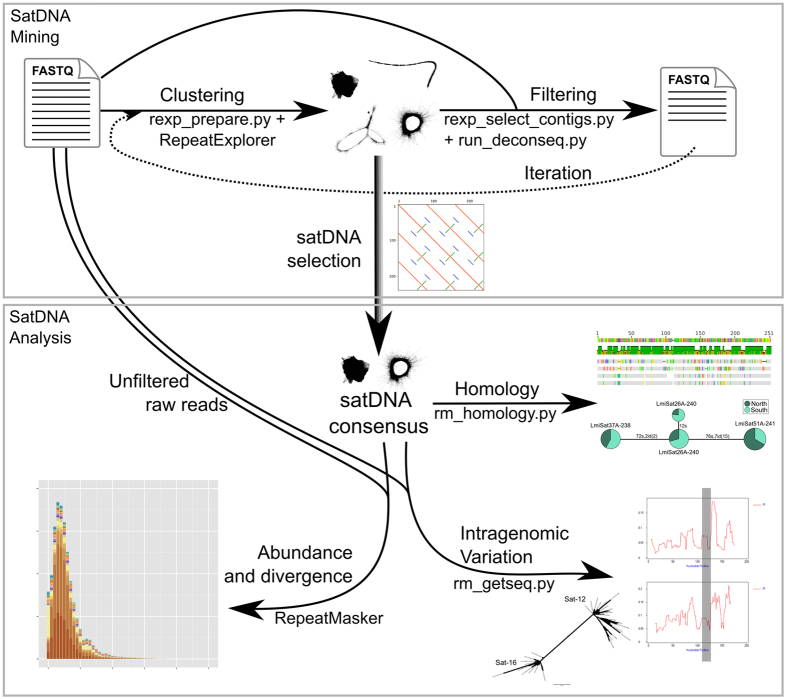
Pipeline for satDNA analysis. The mining steps start with raw reads and a typical clustering with RepeatExplorer. This yields linear, spherical or ring-shaped clusters, the two latter types most likely being satDNAs. Each of these clusters is then split into monomers to search for a consensus satDNA sequence. The assembled sequences, and those showing homology with those included in Repbase and a custom database, were used to filter a new set of raw reads before performing a new RepeatExplorer run. Several clustering and filtering steps were performed until no new satDNA appeared. This increased the number of reads analyzed by Repeat Explorer without greatly increasing computing requirements. The satDNA collection obtained is then analyzed for different features such as homology between different consensus sequences and their intragenomic diversity, and a repeat landscape is built.

We developed satMiner, a toolkit for mining and analyzing satDNA. Scripts and running instructions are freely available in GitHub (https://github.com/fjruizruano/satminer). The satMiner protocol consists of the high-throughput extraction of satDNA sequences and their subsequent analyses. For satDNA mining, we performed a protocol for assembly and identification of satDNA families based on the RepeatExplorer software^10^. Since the number of reads for a RepeatExplorer run is computationally restricted to a few millions, and in order to identify as many satDNA families as possible, our protocol included filtering out reads showing high similarity with previously known sequences (Fig. 6).

The first step consists in discarding low quality reads with Trimmomatic^43^, by removing adapters and selecting read pairs with all their nucleotides, i.e. 2 × 100 or 2 × 101, with Q > 20, using the options “ILLUMINACLIP:TruSeq3-PE.fa:2:30:10 LEADING:3 TRAILING:3 SLIDINGWINDOW:4:20 MINLEN:[100/101]”. We randomly selected 2 × 250,000 reads with SeqTK (https://github.com/lh3/seqtk) and run RepeatExplorer with default options and a custom database of repeated sequences, in addition to Repbase v20.10^44^, last accessed October 28, 2015. We manually selected the clusters with spherical or ring-shaped structure and density values (i. e. the mean number of links per read) being higher than 0.1. For each cluster we chose the contigs showing the highest coverage and generated a dotplot with Geneious v4.8^45^. If we detected tandem structure, we split the contigs in monomers to align them and generate a consensus monomer for each contig. We then chose a new collection of reads and those that matched previously detected satDNAs were filtered out with the DeconSeq v0.4.3 software^46^, with default options, before a new RepeatExplorer run was performed. We used satDNA dimers as reference and, in case of dimers shorter than 200 bp, we concatenated so many monomers as needed to surpass this length. The mismatched reads were then assembled in a new run of RepeatExplorer to search for the presence of satDNAs being poorly represented in the crude reads but detectable in the filtered ones. This procedure increased very much the number of analyzed reads without dramatically increasing computational effort. Therefore, we run RepeatExplorer with 2 × 500,000 filtered reads, searched for new satDNAs and filter them out. We repeated this process two more times adding 2 × 500,000 reads in each iteration, until no new satDNA was detected by RepeatExplorer. We mined satDNAs following the same steps in parallel for the gDNA libraries from the Northern and Southern lineages.

For satDNA sequence analysis, we compared the consensus sequences of all satDNAs found in order to investigate possible homology between some of them. For this purpose, we aligned each satDNA against the whole satDNA catalog with RepeatMasker v4.0.5^47^, using the Cross_match search engine, recording all matches between satDNAs. When sequences showed less than 80% of identity we considered them as different satDNA families sharing a same superfamily. Sequences showing identity higher than 80% were considered variants of the same family, and those showing identity higher than 95% were considered the same variant. We numbered satDNA families in order of decreasing abundance in the Southern lineage individual [GenBank:KU056702–KU056808]. We built a minimum spanning tree for DNA sequences in each superfamily with Arlequin v3.5^48^, considering each indel position as a single change and representing the relative abundance among Southern and Northern individuals.

We used RepeatMasker^47^ with “-a” option to estimate abundance and divergence for each satDNA variant in gDNA libraries. We selected 2 × 5 millions of paired reads where all nucleotides met quality criteria applied for the satMiner protocol. Abundance estimates provided by RepeatMasker showed highly significant positive correlation with those yielded by RepeatExplorer in both the Southern (Spearman r_s_ = 0.84, N = 15, P = 0.000074) and Northern (r_s_ = 0.97, N = 17, P < 0.000001) lineages. Compared to RepeatExplorer, RepeatMasker has the advantage of working on a much higher number of reads, with reasonable computing times, and it can simultaneously estimate the abundance of all satDNA variants previously collected, whereas several runs of RepeatExplorer are necessary to obtain the whole collection of satDNAs, using different reads thus making it difficult normalization, especially for rare variants. We estimated the average divergence generating a repeat landscape considering distances from the sequences applying the Kimura 2-parameter model with the script calcDivergenceFromAlign.pl within the RepeatMasker suite^47^. In the resulting output, we calculated the weighed mean divergence for each satDNA family, considering all variants. Additionally, we estimated each satDNA family abundance as the sum of nucleotides for all variants. We normalized per the number of selected nucleotides and represented abundance as percentage of the library, i.e., genome proportion.

We estimated the frequency of random occurrence for some short satDNA monomers. For this purpose, we generated 1,000 Gb, i.e., ~159 genomes, shuffling nucleotides with the uShuffle program^49^ preserving the dinucleotide frequencies of the assembled genome of *L. migratoria*^11^, accession number AVCP000000000. In addition, we analyzed the abundance of some satDNA families in these artificial genomes by using RepeatMasker^47^. For each satDNA family, we scored the number of contigs where at least 200 bp were present, and also scored the number of nucleotides aligning in each contig.

### Primer design and PCR

We tested the reliability of the satDNAs found and synthtesized FISH DNA-probes by PCR amplification of all satDNA families. For this purpose, we aligned each satDNA monomers to get a consensus sequence and selected the most conserved region to design primers in opposite orientation ensuring to minimize the distance between them or even overlapping them up to 3 bp at the 5′ end, when necessary (Supplementary Table S8, Supplementary Fig. S7a). For this purpose, we used the Primer3 software^50^ with an optimal melting temperature of 60 °C. Alternatively, for monomers shorter than 50 bp, we designed primers manually with a similar melting temperature and with the less stable extensive dimers predicted by the software PerlPrimer^51^ (Supplementary Fig. S7b). For families with monomer longer than 50 bp, we performed PCR amplification with a starting denaturation step of 95 °C during 5 min, 35 cycles with 94 °C during 20 s, with 55–65 °C as annealing temperature during 40 s and 72 °C during 20 s and a final extension step of 7 min. We checked the resulting products in a 2% agarose gel to see the typical ladder pattern of tandem repeats (Supplementary Fig. S7a). We trimmed the band of the monomer for the annealing temperature with less smear and extracted the DNA squeezing it in a parafilm square. We reamplified 0.5 μL of the resulting solution. For satDNAs shorter than 50 bp, we reduced the time of annealing to 10 s in order to get longer amplicons. This PCR displayed a smear (Supplementary Fig. S7b). We performed a reamplification using 0.2 μL of the previous PCR product. We purified all PCR products using the GenElute PCR Clean Up kit (Sigma). We only got success for 59 satDNAs, 25 of which were Sanger sequenced and the reliability of the PCR product was confirmed.

### Physical mapping

All these PCR products were labeled by nick translation with 2.5 units of DNA polymerase I/DNase I (Invitrogen), following the standard protocol, to be used as DNA probes for fluorescent *in situ* hybridization (FISH). Mapping of satDNAs was performed following the protocol described in Cabrero *et al*.^52^. FISH probes were labeled with tetramethylrhodamine-5-dUTP (satDNAs) or fluorescein-12-dUTP (rDNA and histone H3 genes) from Roche.

*L. migratoria* chromosomes are all acrocentric and the autosomes can be classified into three size groups: long (L1 and L2), medium (M3–M8) and short (S9–S11). The X chromosome is the third element in size. Previous research has shown that, in this species, the 45S ribosomal DNA (rDNA) is distally located on L2 and M6, and interstitially on S9 chromosomes^53^, whereas a single histone gene cluster is interstitially located on M8^54^. We employed these two markers to perform double FISH with selected satDNAs located on M chromosomes scarcely differing in size, to identify the satDNA-carrying chromosome. In addition, it is known that the L1 autosome carries the U1 snRNA gene cluster^55^, but we differentiated this chromosome from L2 because the latter carries a distal cluster of rDNA. We distinguished three types of satDNA localization: proximal to centromere in any chromosome arm (p), interstitial in the long arm (i) and distal to centromere in the long arm (d). Most FISH analyses were performed on embryo preparations made following Camacho *et al*.^14^.

### Statistical analyses

Statistical analyses included non parametric Spearman rank correlation, Wilcoxon matched pairs test, Wilcoxon one-sample test and Mann-Whitney test, all of them performed with Statistica soft. Contingency chi-square tests were performed with the RXC program (George Carmody, University of Ottawa, Canada) by a Monte Carlo approach to calculate statistical significance, with 5,000 permutations. When multiple tests were performed, the resulting probability was corrected by the sequential Bonferroni method^56^, represented here as Pb.

## Additional Information

**How to cite this article**: Ruiz-Ruano, F. J. *et al*. High-throughput analysis of the satellitome illuminates satellite DNA evolution. *Sci. Rep.* **6**, 28333; doi: 10.1038/srep28333 (2016).

## Supplementary Material

Supplementary Information

## Figures and Tables

**Table 1:** Length (nt), A + T content (%), number of variants (V), abundance (% of the genome), divergence (%), number of contigs found in the draft genome of *Locusta migratoria* 11, maximum number of repeats per contig (MNRPC), chromosome location (in the Southern lineage) and clustering pattern of all 62 satDNA families and superfamilies (SF).

SF	SatDNA Family	Length	A + T	V	Abundance		Divergence		*L. migratoria* genome (NL)^11^		Chromosome location (SL)												Pattern
					SL	NL	SL	NL	Contigs	MNRPC	L1	L2	X	M3	M4	M5	M6	M7	M8	S9	S10	S11	
1	LmiSat01–193	193	59.59	5	0.98225	0.6903	4.67	5.07	332	15	p	p	p	p	p	p	p	p	p	p	p	p	c
	LmiSat02–176	176	53.41	1	0.47509	0.9996	5.32	5.38	12931	100			p			p		p		p	p	p	c
	LmiSat03–195	195	58.97	6	0.29481	0.2305	5.42	5.96	1003	206	p			p									c
	LmiSat04–18	18	50	2	0.06194	0.0816	7.2	7.23	108	156										i,d		i	c
	LmiSat05–400	400	51.25	1	0.05431	0.0483	4.65	5.04	91	3										i,d		p	c
	LmiSat06–185	185	59.46	4	0.0541	0.07	4.76	5.28	274	42		p		p					p	p		p	c
	LmiSat07–5-tel	5	60	1	0.04438	0.1611	1.75	6.12	57	2868	t	t	t	t	t	t	t	t	t	t	t	t	c
	LmiSat08–168	168	57.74	1	0.03737	0.0467	4.96	4.91	327	28			p										m
	LmiSat09–181	181	60.22	5	0.02944	0.0072	5.38	7.42	45	60									p				c
	LmiSat10–9	9	55.56	2	0.02269	0.029	11.79	11.42	267	243										p		p	c
	LmiSat11–37	37	62.16	7	0.01873	0.0069	7.75	8.12	317	106				p									c
2	LmiSat12–273	273	56.41	3	0.01836	0.0113	3.5	5.29	23	16		d											c
1	LmiSat13–259	259	57.53	5	0.01697	0.0115	4.38	6.25	137	27					p								c
	LmiSat14–216	216	51.85	4	0.01426	0.0091	5.39	8.79	70	40				i,i								i	c
	LmiSat15–190	190	55.26	1	0.01426	0.0166	4.09	4.5	212	9								p					c
2	LmiSat16–278	278	62.59	1	0.0139	0.0082	2.49	3.01	17	9		d											c
	LmiSat17–75	75	57.33	1	0.01177	0.0033	5.79	6.66	112	7				p									c
	LmiSat18–210	210	60.48	1	0.01121	0.0267	6.33	4.59	6	2								p					c
	LmiSat19–89	89	60.67	1	0.01058	0.0034	3.82	6.44	10	4		p											c
	LmiSat20–15	15	53.33	1	0.01032	0.0201	12.71	14.15	190	256													nc
	LmiSat21–38	38	50	1	0.01013	0.0019	2.85	2.91	7	20										i,d			m
	LmiSat22–17	17	58.82	1	0.01	0.0092	10.81	10.28	182	426								i					c
	LmiSat23–223	223	61.43	1	0.00927	0.0106	4.42	5.73	18	10		d					d			i			c
3	LmiSat24–266	266	56.39	1	0.00895	0.0066	2.06	5.14	51	4													nc
	LmiSat25–219	219	39.73	2	0.00834	0.0105	5.88	8.2	21	5		d											c
4	LmiSat26–240	240	66.2	2	0.00809	0.00436	7.44	9.25	33	8												i	c
	LmiSat27–57	57	47.37	1	0.0079	0.0103	8.99	9.66	333	326													nc
3	LmiSat28–263	263	57.41	2	0.00768	0.0139	1.79	2.22	91	12		i,i											c
	LmiSat29–68	68	58.82	1	0.00719	0.0019	9.36	14.48	46	89				p									c
	LmiSat30–138	138	40.58	1	0.0068	0.0055	5.74	9.03	8	2									p				c
5	LmiSat31–8	8	50	3	0.00668		3.86		23	83										p	p		c
	LmiSat32–261	261	51.72	1	0.00631	0.0056	5.98	9.18	37	12		d											c
	LmiSat33–21	21	47.62	1	0.00627	0.0039	7.77	8.35	30	179										i			c
	LmiSat34–299	299	61.87	1	0.00622	0.0048	6.81	7.39	406	3		p											c
	LmiSat35–228	228	55.7	1	0.00597	0.0053	2.43	4.64	25	18													nc
	LmiSat36–15	15	60	2	0.00585	0.0093	16.88	15.12	279	302													nc
4	LmiSat37–238	238	66	1	0.00544	0.00224	6.53	6.52	111	37	i											p	c
	LmiSat38–42	42	64.29	1	0.00511	0.0046	14.56	14.94	106	692												i	c
	LmiSat39–53	53	32.08	1	0.00503	0.0013	6.79	9.17	14	119										i			c
	LmiSat40–148	148	67.57	1	0.00459	0.0023	2.35	3.05	20	4										d			c
	LmiSat41–180	180	61.67	1	0.00455	0.0058	3.38	2.14	4	6									i				c
	LmiSat42–127	127	51.18	1	0.00447	0.0012	2.02	4.6	2	2						p							c
3	LmiSat43–231	231	53.68	1	0.0044		0.68		44	3									i				m
	LmiSat44–17	17	29.41	1	0.00428	0.0005	11.45	11.3	7	53										i			c
3	LmiSat45–274	274	54.01	1	0.0042	0.0066	8.2	7.22	152	12	p,i		p	p									c
	LmiSat46–353	353	59.77	1	0.00407	0.0071	15.49	11.38	1799	2													
	LmiSat47–41	41	41.46	1	0.00369	0.0058	12.46	13.22	48	394					p								c
	LmiSat48–220	220	58.18	1	0.00366	0.0011	3.8	7.74	18	3													nc
	LmiSat49–47	47	42.55	1	0.00362	0.0113	6.24	6.7	127	282				p									c
5	LmiSat50–16	16	56.25	2	0.00331	0.0169	8.31	8.24	54	64										i			c
4	LmiSat51–241	241	63.9	1	0.00294	0.0058	7.32	3.97	33	138		i											c
	LmiSat52–143	143	51.75	1	0.00257	0.0076	22.15	14.01	1796	3													
	LmiSat53–47	47	40.43	1	0.00248	0.019	3.16	5.2	9	23										i			c
3	LmiSat54–272	272	56.25	1	0.00244	0.0051	4.55	4.15	164	51			p	i			i	p	d				m
	LmiSat55–90	90	35.56	1	0.00164	0.0074	15.62	8.57	4	3													nc
	LmiSat56–19	19	52.63	4	0.00083	0.0067	5.09	4.31	15	97							p			i			m
	LmiSat57–230	230	63.04	1	0.00052	0.0047	18.21	3.4	212	25													
	LmiSat58–86	86	41.86	1	0.00008	0.0127	5.99	3.12	10	4													nc
5	LmiSat59–16	16	43.75	3	0.00004	0.0049	18.23	14.54	13	13													nc
	LmiSat60–255	255	52.94	1	0.00004	0.0053	1.03	0.99	0	0													nc
	LmiSat61–63	63	42.86	1	0.00002	0.0062	14.99	4.6	1	11													nc
	LmiSat62–23	23	43.48	1		0.0045		4.57	1	9								p					c
	Total			107	2.39241	2.7417				Total p	3	4	5	8	3	3	2	6	4	5	3	6	52
										Total i	2	3	0	3	0	0	1	1	2	10	0	4	26
										Total d	0	5	0	0	0	0	1	0	1	4	0	0	11
										Total loci	5	12	5	11	3	3	4	7	7	19	3	10	89
										satDNAs	4	11	5	10	3	3	4	7	7	16	3	10	83

In each family, length and A + T content are given for the most abundant variant. Divergence per family is expressed as percentage of Kimura divergence. Chromosome location was analyzed by FISH in a Spanish population. SL = Southern lineage, NL = Northern lineage. Chromosome locations: t = telomeric, p = proximal to centromere, i = interstitial, d = distal. Chromosome distribution patterns: c = clustered, nc = non-clustered, m = mixed. When a satDNA showed two loci in a same chromosome, their locations were indicated separated by a comma. Totals at the bottom do not include LmiSat07–5 (the telomeric repeat).
