# Supplementary material for: High-throughput analysis of the satellitome illuminates satellite DNA evolution
Source: Sci Rep. 2016 Jul 7;6:28333. doi: 10.1038/srep28333 (PMC4935994; doi:10.1038/srep28333)
Supplement: Supplementary Information [file srep28333-s1.pdf]

Supplementary Information

High-throughput analysis of the satellitome illuminates satellite DNA evolution

Francisco J. Ruiz-Ruano, María Dolores López-León, Josefa Cabrero and Juan Pedro M. Camacho

Departamento de Genética, Facultad de Ciencias, Universidad de Granada, Granada, Spain

Contents

Supplementary Results **3**

Results S1 | Northern and Southern lineage genomes show very similar satellitome content 3

Results S2 | Monomer length variation 3

Results S3 | Chromosome location 4

Supplementary Figures **5**

Figure S1 | Alignments between the different variants of satDNA families 5

Figure S2 | Repeat landscapes in Southern and Nothern lineages 6

Figure S3 | Minimum spanning trees for superfamilies 1, 2, 4 and 5 7

Figure S4 | Alignment of the sequences belonging to superfamily 5 9

Figure S5 | Alignments of all satDNAs matching with Repbase entries 10

Figure S6 | Phylogeny for full mitogenomes 11

Figure S7 | Primer design and PCR amplification 12

Supplementary Tables **13**

Table S1 | SatDNA families reported in Orthoptera before this study 13

Table S2 | Genomic characterization of satDNA variants 14

Table S3 | Number of chromosome-specific satDNA families 17

Table S4 | Calculation of the equilocality index 18

Table S5 | Extremely short satDNAs can arise by chance in the huge genome of *L. migratoria* 19

Table S6 | Homology of *L. migratoria* satDNAs with other Orthoptera sequences in Repbase 20

Table S7 | Characterization of the *Luzula elegans* satellitome 21

Table S8 | Primers designed in this study to amplify each satDNA family 24

Table S9 | Frequency of repeats of different lengths observed in the simulated *L. migratoria* genomes 24

References **26**

Supplementary Results

Results S1 | Northern and Southern lineage genomes show very similar satellitome content

A comparison of satDNA abundance between the Southern and Northern genomes showed good general agreement in abundance (Spearman rank correlation: $r_S = 0.50$, $N = 58$, $t = 4.34$, $P = 0.000059$; Wilcoxon matched pairs test: $T = 807$, $P = 0.71$). Likewise, satDNA divergence in the 55 satDNAs found in both genomes showed significant positive correlation ($r_s = 0.76$, $t = 8.45$, $P < 0.000001$), but it showed a significant tendency to be higher in the Northern genome ($T = 472.5$, $P = 0.013$). It is necessary to bear in mind that our analyses were made in a single individual per lineage, thus being intragenomic but not population estimates. Therefore, we cannot rule out that a given satDNA being absent in one of the two genomes analyzed might actually be present in other individuals from the same lineage. For instance, LmiSat62-23 was not bioinformatically found in the Southern genome, but it was observed by FISH in a different individual belonging to this same lineage (see Table 1).

Results S2 | Monomer length variation

The 58 satDNAs showed high variation for monomer length (8-400 nt) and A+T content (29.4-67.6%) (Table 1), two parameters showing significant positive correlation ($r_S = 0.35$, $t = 2.8$, $P = 0.006$) thus suggesting that longer satDNAs tend to show higher A+T content and shorter ones tend to be G+C rich. Monomer length distribution showed a bimodal distribution, with a 37 nt gap (between 90 and 127 nt) dividing the 58 satDNAs into two groups, one including 26 short satDNAs (8-90 nt) and the other comprising 32 long satDNAs (127-400 nt). A comparison of A+T content between both groups showed significantly higher A+T content in the long satDNAs (Mann-Whitney test: $U = 214.5$, $P = 0.0016$), confirming the tendency suggested by the Spearman rank correlation above. Remarkably, the A+T average for the 58 satDNAs (53.44%) was significantly lower than that in the L. migratoria genome (59.32%) (Wilcoxon one-sample test: $T = 290$, $P = 0.000012$). The same bias was apparent when compared with Wilmore and Brown's[1] estimate of 58.37% A+T for the whole genome of this species ($T = 392$, $P = 0.0003$). This suggests that satDNAs in this species tend to arise from G+C-rich regions, which is more evident for short (mean= 49.17%; $T = 22$, $N = 26$, $P =$

0.000097) than long (mean= 56.91%; T= 160, N= 32, P= 0.052) ones. In addition, short satDNAs showed higher divergence than long ones (Southern genome: U= 132, P= 0.000016; Northern genome: U= 193.5, P= 0.0014). Taken together, these results indicate that short satDNAs show higher divergence and G+C content than long ones.

Results S3 | Chromosome location

The frequencies of clustered, non-clustered and mixed patterns (17, 7 and 2, respectively, for short satDNAs, and 25, 4 and 3, respectively, for long ones) did not differ significantly between the two length classes (RxC with 50,000 replicates: P= 0.343, SE= 0.006).

The total number of proximal, interstitial and distal loci did not differ significantly between short and long satDNAs (RxC: P= 0.170, SE= 0.006).

Supplementary Figures

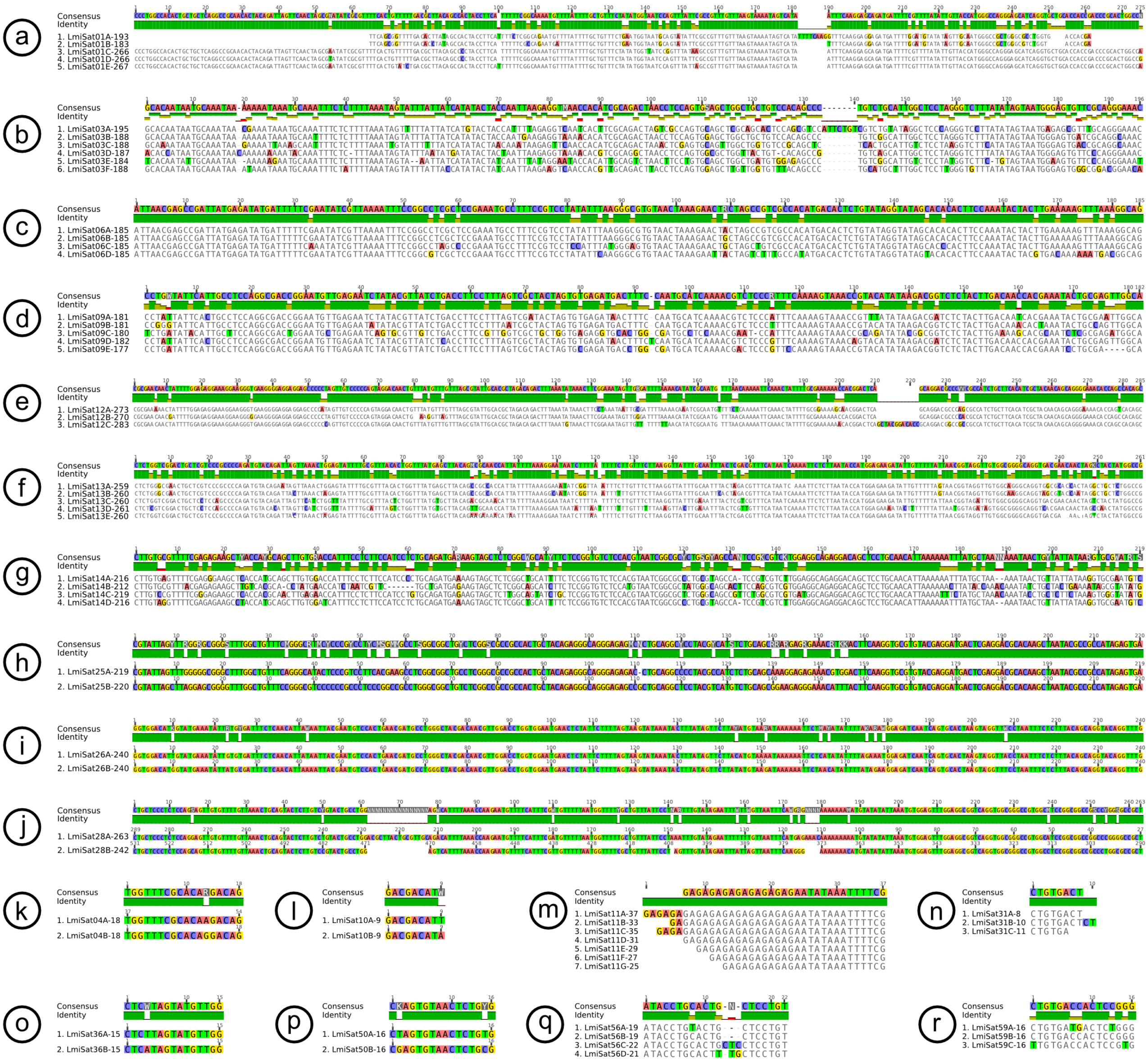

Figure S1: Alignments between the different variants found for several long (**a**-**j**) and short (**k**-**r**) satDNA families.

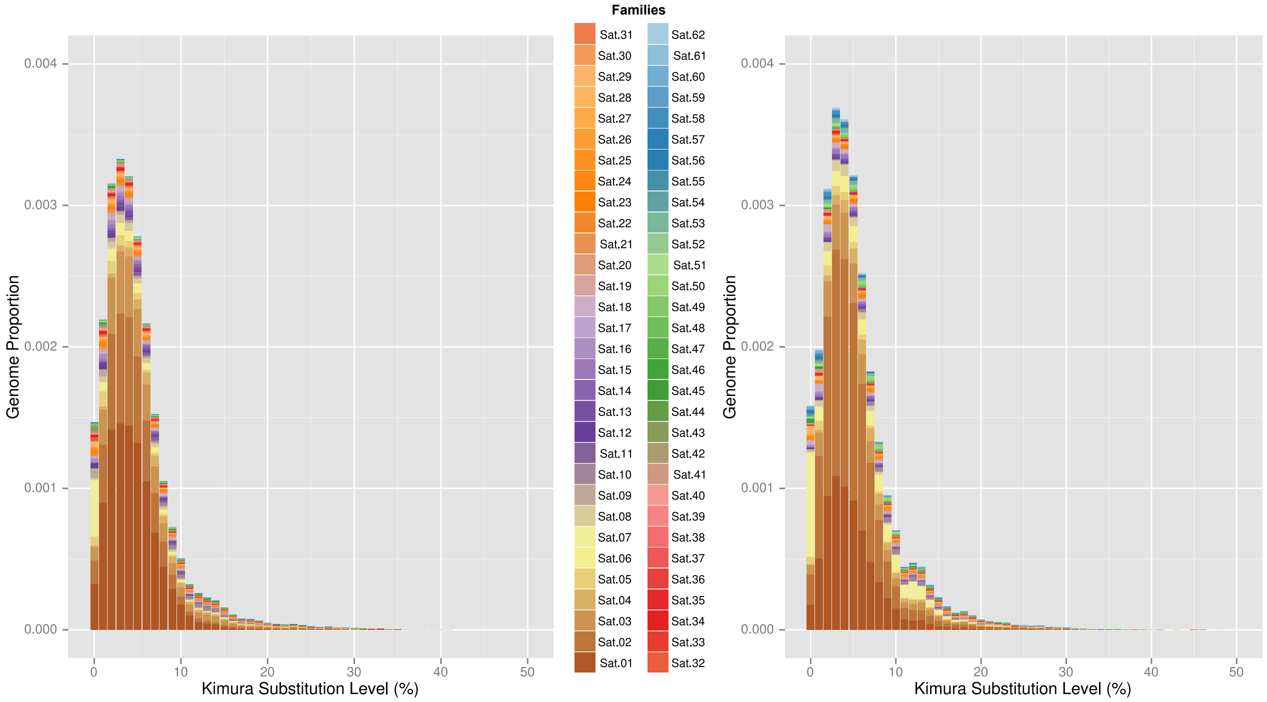

Figure S2: Repeat landscapes for the 62 satDNA families in the individuals analyzed from the Southern (left) and Northern (right) lineages. Note that both lineages show a similar collection of satDNAs with only slight variations in abundance.

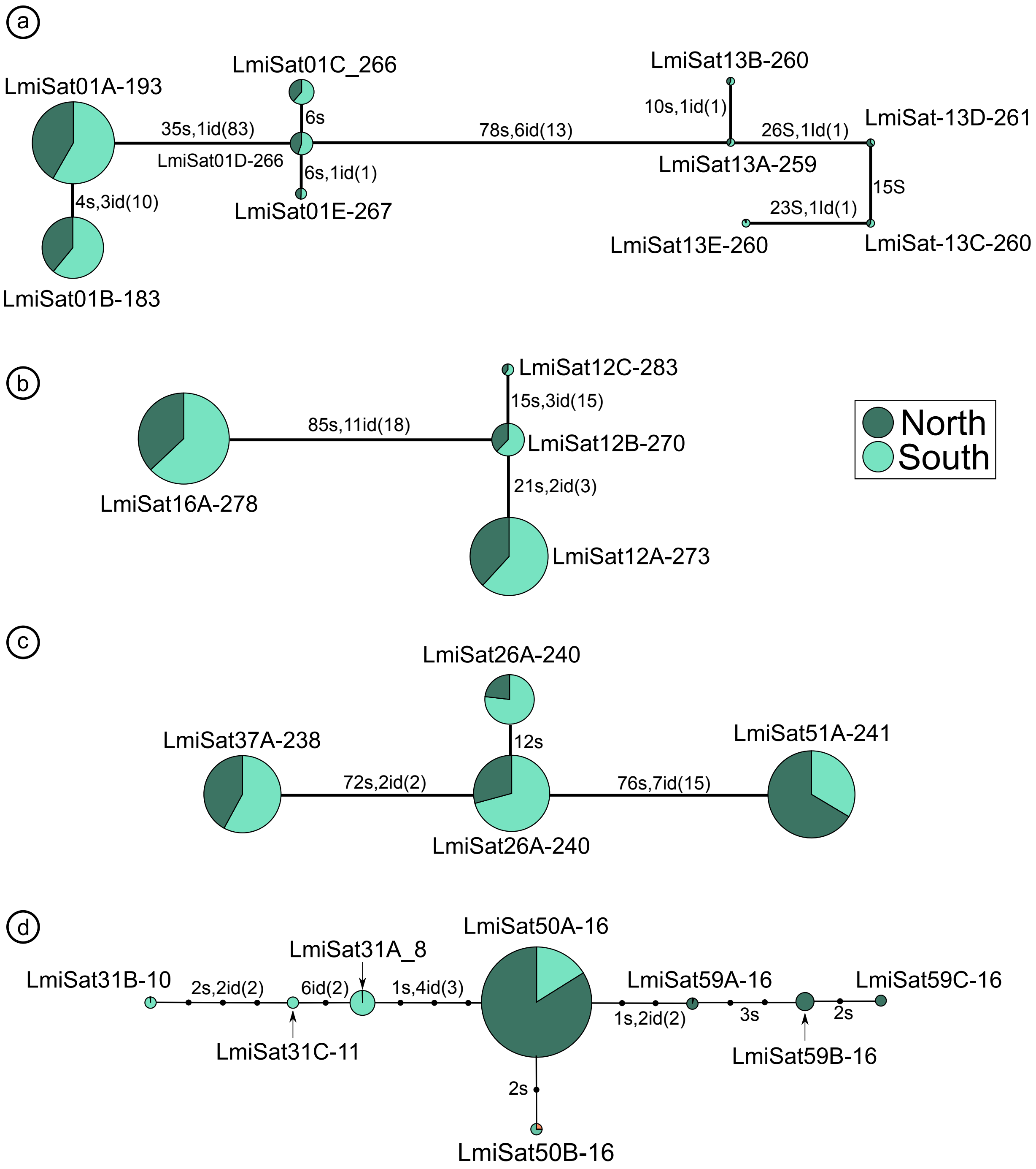

Figure S3: Minimum spanning trees for superfamilies 1, 2, 4 and 5 (**a**-**d**). In **a**-**c**, link size between haplotypes is proportional to the number of substitutions (s) and indels (id) (in **d**, links are also indicated as mutational steps). In brackets is indicated the sum of nucleotides involved in the indels. [Legend continues in the next page]

Figure S3 [Continuation]: **a**) Superfamily 1 (SF1) includes five sequence variants for LmiSat01-193 (three showing lengths about 1.5 fold the two remainder) and five for LmiSat13-259 (showing lengths similar to those of the longest LmiSat01-193 variants). On the basis of abundance, the ancestral monomer for this superfamily might be about 180-190 nt long (LmiSat01A-193 and LmiSat01B-183 variants), and the remaining variants in SF1, which are about 260 nt long, arose through a 83 nt insertion. Both satDNA families locate pericentromerically, but LmiSat01-193 was on all chromosomes and LmiSat13-259 was only on M4 (Table 1), suggesting that LmiSat13-259 arose from LmiSat01-193 in the M4 chromosome. **b**) Superfamily 2 (SF2) includes three sequence variants for LmiSat12-273 (270-283 nt) and one for LmiSat16-278 (278 nt). The exclusive presence of these two satDNAs at a coincident distal location in the L2 chromosome suggests that SF2 arose in this chromosome and has not moved to other non-homologous chromosomes. This case illustrates how the differential accumulation between variants give rise to new satDNA families when similarity decreases beyond the 80% criterion. **c**) Superfamily 4 (SF4) includes two variants of LmiSat26-240 (240 nt) and a single variant of LmiSat37-238 and LmiSat51-241. All three satDNA families were interstitially located but on different chromosomes: S11, L1 and L2, respectively, with LmiSat37-238 showing a second cluster proximally located on S11. SF4 thus reflects how satDNAs move between non-homologous chromosomes. **d**) Superfamily 5 (SF5) included three short satDNAs (LmiSat31-8, LmiSat50-16 and LmiSat59-16) showing different location patterns: LmiSat31-8 is pericentromeric on S9 and S10, LmiSat50-16 is interstitial on S9, and LmiSat59-16 is non-clustered. Sequence alignment suggests that LmiSat49-16 and LmiSat58-16 families could have arisen from LmiSat31-8 through duplication (Supplementary Fig. S4). However, a minimum spanning tree for these three families suggests that LmiSat50A-16 (which is abundant in both lineages) is the ancestral variant, and that LmiSat31-8 emerged in the Southern genome and LmiSat59-16 in the Northern one. In addition, the fact that simulated genomes of *L. migratoria* would contain, by chance, more than 200,000 copies of DNA motives identical to the three LmiSat31-8 variants (Supplementary Table S5), together with its exclusive presence in the Southern genome, suggests the possibility that this extremely short satDNA arose independently from the two other SF5 members in the Southern lineage. Likewise, LmiSat50-16 and LmiSat59-16 could represent a case of derivation of LmiSat59-16 from LmiSat50-16 in the Northern lineage, but the fact that simulated genomes included 6 and 4 copies, respectively, for both (Supplementary Table S5), and their different patterns of chromosomal location (clustered and non-clustered, respectively) throw some doubts on this possibility. Therefore, the reliability of SF5 needs additional analysis.

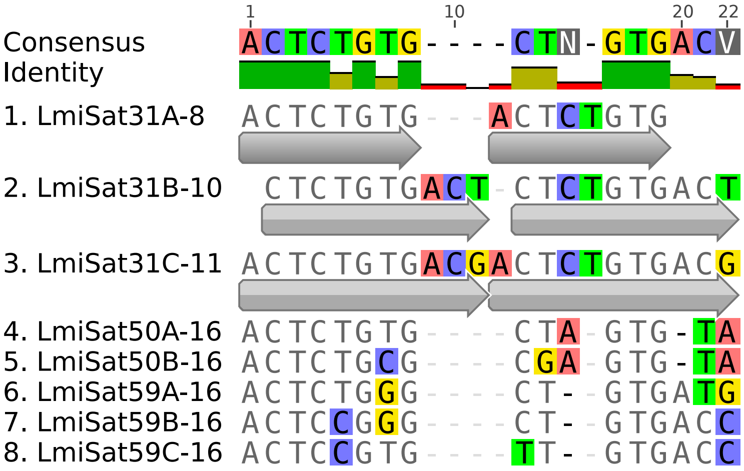

Figure S4: Alignment of LmiSat31-8 dimers and LmiSat50-16 and LmiSat59-16 dimers, all belonging to superfamily 5, showing how the two latter families could have derived from a dimer for the former satDNA.

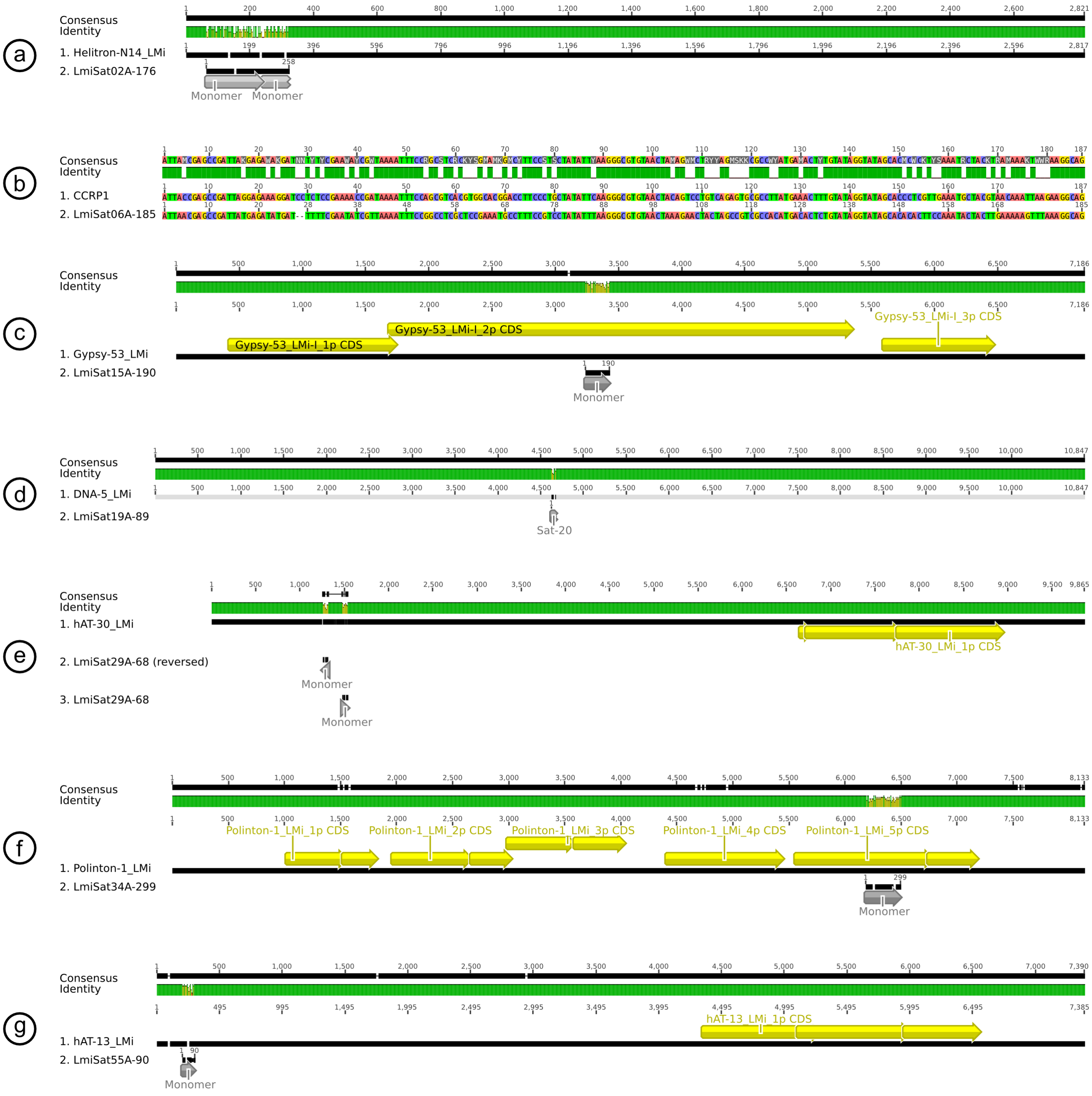

Figure S5: Alignments of all satDNAs matching with Repbase entries.

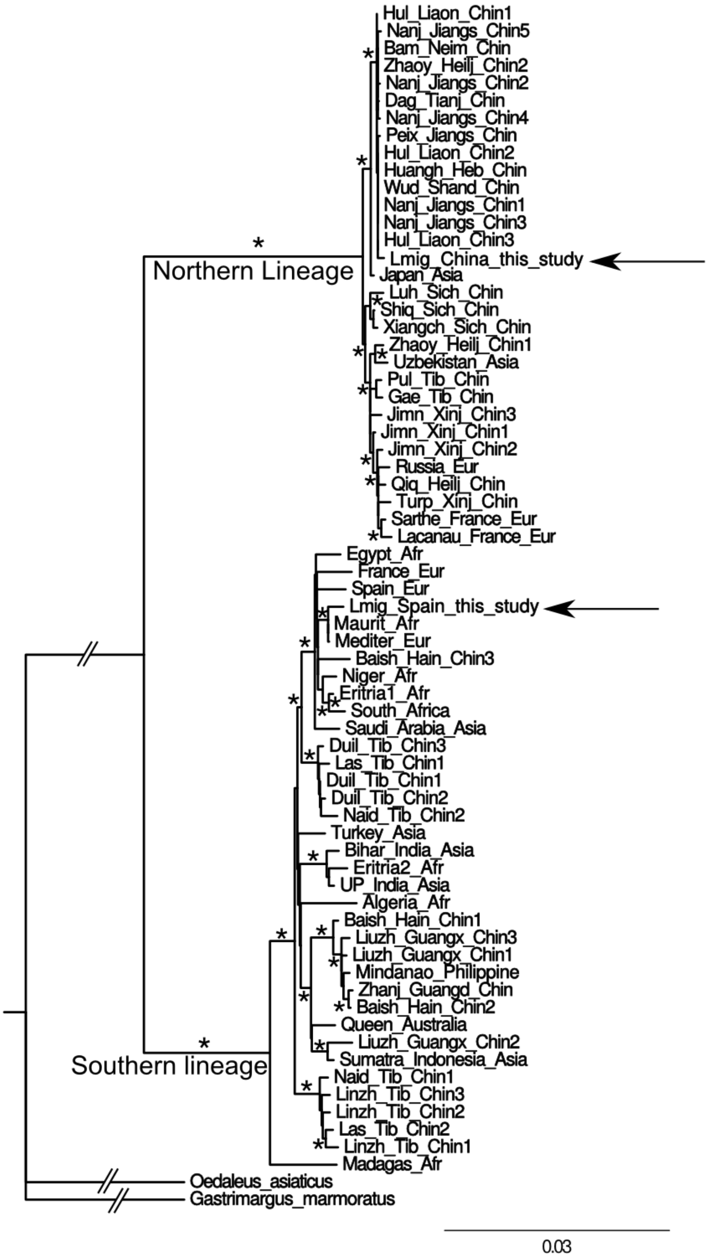

Figure S6: Maximum likelihood phylogeny for full mitogenomes reported by Ma et al.[2] in *L. migratoria* and those assembled by us from the same Illumina reads used to search for satDNAs in this study, from a Spanish and a Chinese individuals (arrows). Asterisks indicate branch supports higher than 90%. Note that the Spanish individual clustered with Southern mitogenomes whereas the Chinese one corresponds to the Nothern lineage.

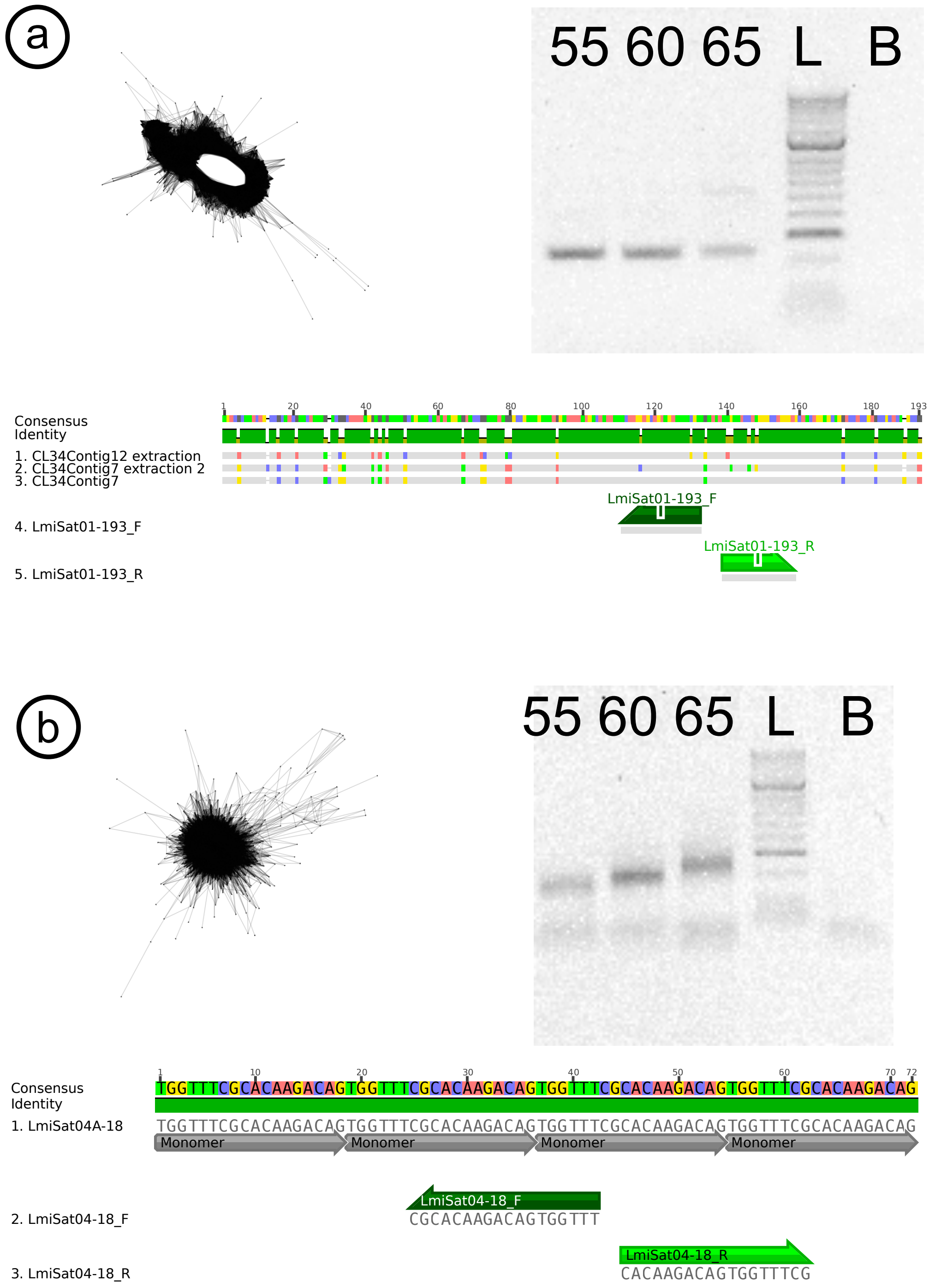

Figure S7: Primer design and PCR amplification for long (**a**) and short (**b**) satDNAs. Note that long satDNAs (e.g. LmiSat01-193 shown here) show ring-shaped RepeatExplorer cluster graphs because read length is lower than monomer length. We designed divergent primer pairs, with nearby 5′ ends, and tested them at 55, 60 and 65ºC annealing temperature. Dimer amplification was manifested at the highest temperature (**a**). Short satDNAs (e.g. LmiSat04-18 shown here) show spherical RepeatExplorer cluster graphs because monomer length is lower than read length. We designed divergent primers with the less stable extensive dimers. We obtained a delimited smear showing higher size with increasing annealing temperature (**b**).

Supplementary Tables

Species	Source	Name	nt	Method	Characteristics
Warramaba virgo	[3]	–	–	CoT	–
Atractomorpha similis	[4]	537bp	537	Restriction (TaqI)	–
Caledia captiva	[5]	168bp	168	Restriction (TaqI)	Iinterstitial and distal
	[6]	144bp	144	Restriction (TaqI)	Pparacentromeric
Stauroderus scalaris	[7]	168bp	168	CoT	Not determined (probably distal)
Dociostaurus genei	[8]	DgT2	160	Restriction (TaqI)	Centromeric C-bands in each chromosome of the complement
	[8]	DgA3	217	Restriction (AluI)	Distal C-bands present in most of the autosomal pairs
Dolichopoda spp.	[9]	pDoP102	102	Restriction (PstI)	Species specific for *D. schiavazzii*, 30% of the genome
	[10]	pDsPv400	~400	Restriction (PvuII)	Species specific for *D. schiavazzii*
	[10]	pDoP500	~500	Restriction (PstI)	Probably present in all *Dolichopoda* species. (*D. laetitia laetitia*)
Eyprepocnemis plorans	[11]	180bp	180	Restriction (DraI)	Paracentromeric and B chromosome
Oxya hyla intricata	[12]	169bp	169	Restriction (HaeIII)	C-bands of the short arms of most of the chromosomes. Species-specific
	[12]	204bp	204	Restriction (HaeIII)	Centromeric in three chromosome pairs. Specific of *O. hyla intricata*
Gryllus bimaculatus	[13]	GBH535	535	Restriction (HindIII)	Conserved in *Gryllus* species. Derived from a common ancestral sequence
	[13]	GBH542	542	Restriction (HindIII)	Species-specific
Arcyptera fusca	[14]	EcoRV-390CEN	390	W-CGH	Centromeric
and *Arcyptera tornosi*	[14]	Sau3A-419CEN	419	W-CGH	Centromeric
	[14]	Sau3A-197TEL	197	W-CGH	Heterochromatic distal regions
Schistocerca gregaria	[15]	SG1	171	NGS	Pericentromeric regions of complement
	[15]	SG2-alpha	352	NGS and Rest. (HindIII)	Distal C-bands of the three shortest chromosomes
	[15]	SG3	170	NGS	Interstitially in chromosome S10

Table S1: SatDNA families reported in Orthoptera before this study. NGS: Next-Generation Sequencing. W-CGH: Whole-Comparative genomic hybridization.

			Abundance		Repeats		Divergence	
Variant	Length	A+T	SL	NL	SL	NL	SL	NL
LmiSat01A	193	59.59	0.39467	0.28298	128830	92373	5.21	5.52
LmiSat01B	183	60.11	0.30928	0.19809	106473	68194	3.89	4.05
LmiSat01C	266	56.02	0.12652	0.07954	29966	18840	2.89	3.11
LmiSat01D	266	55.64	0.10516	0.08471	24907	20063	6.89	7.62
LmiSat01E	267	56.55	0.04661	0.04494	10998	10605	4.61	5.23
LmiSat02A	176	53.41	0.47509	0.99959	170059	357809	5.32	5.38
LmiSat03A	195	58.97	0.21447	0.17144	69290	55390	4.25	4.68
LmiSat03B	188	60.64	0.02336	0.02088	7828	6997	11.97	12.89
LmiSat03C	188	62.77	0.02219	0.01716	7437	5750	6.53	7.60
LmiSat03D	187	62.03	0.01625	0.01140	5475	3841	10.27	10.64
LmiSat03E	184	63.59	0.00963	0.00397	3296	1361	4.89	4.78
LmiSat03F	188	62.77	0.00891	0.00564	2987	1889	5.08	4.81
LmiSat04A	18	50.00	0.05540	0.07290	193895	255147	6.66	6.80
LmiSat04B	18	44.44	0.00654	0.00869	22877	30408	11.82	10.87
LmiSat05A	400	51.25	0.05431	0.04827	8553	7603	4.65	5.04
LmiSat06A	185	59.46	0.01845	0.01558	6284	5307	4.62	5.59
LmiSat06B	185	60.00	0.01759	0.02093	5991	7128	5.49	5.29
LmiSat06C	185	59.46	0.01625	0.02317	5535	7889	3.38	4.23
LmiSat06D	185	61.08	0.00180	0.01034	612	3522	10.93	7.14
LmiSat07A	5	60.00	0.04438	0.16113	559196	2030244	1.75	6.12
LmiSat08A	168	57.74	0.03737	0.04669	14015	17510	4.96	4.91
LmiSat09A	181	60.22	0.01405	0.00252	4892	876	1.07	1.53
LmiSat09B	181	58.01	0.00554	0.00179	1929	622	6.03	6.27
LmiSat09C	180	49.44	0.00419	0.00070	1468	245	14.57	21.06
LmiSat09D	182	58.24	0.00408	0.00127	1411	439	7.17	8.40
LmiSat09E	177	53.11	0.00157	0.00098	560	348	10.98	12.200
LmiSat10A	9	55.56	0.02052	0.02700	143648	189028	11.62	11.23
LmiSat10B	9	55.56	0.00217	0.00200	15172	14028	13.27	13.88
LmiSat11A	37	62.16	0.00651	0.00222	11082	3784	7.69	7.46
LmiSat11B	33	63.64	0.00342	0.00090	6538	1709	7.81	8.34
LmiSat11C	35	62.86	0.00315	0.00150	5677	2695	8.30	8.25
LmiSat11D	31	64.52	0.00301	0.00125	6114	2535	7.64	8.17
LmiSat11E	29	65.52	0.00145	0.00052	3157	1128	6.98	9.11
LmiSat11F	27	66.67	0.00090	0.00034	2104	789	7.81	8.52
LmiSat11G	25	68.00	0.00028	0.00021	705	529	7.63	10.03
LmiSat12A	273	56.41	0.01170	0.00723	2701	1669	2.19	3.64
LmiSat12B	270	53.33	0.00494	0.00297	1152	694	5.77	7.23
LmiSat12C	283	53.71	0.00172	0.00112	382	250	5.52	10.50
LmiSat13A	259	57.53	0.00723	0.00488	1758	1188	4.01	6.40
LmiSat13B	260	59.23	0.00596	0.00443	1444	1074	4.44	5.20
LmiSat13C	260	62.69	0.00292	0.00196	707	475	5.81	7.36
LmiSat13D	261	65.13	0.00073	0.00005	177	12	1.78	21.24
LmiSat13E	260	62.69	0.00013	0.00022	32	52	6.04	9.97

			Abundance		Repeats		Divergence	
Variant	Length	A+T	SL	NL	SL	NL	SL	NL
LmiSat14A	216	51.85	0.00584	0.00468	1703	1365	11	69
LmiSat14B	212	51.89	0.00441	0.00257	1312	763	5.81	7.34
LmiSat14C	219	50.23	0.00241	0.00137	693	393	47	79
LmiSat14D	216	53.24	0.00160	0.00048	467	140	3.62	10.65
LmiSat15A	190	55.26	0.01426	0.01660	4727	5504	09	50
LmiSat16A	278	62.59	0.01390	0.00817	3149	1851	2.49	3.01
LmiSat17A	75	57.33	0.01177	0.00335	9891	2810	79	66
LmiSat18A	210	60.48	0.01121	0.02669	3362	8008	6.33	4.59
LmiSat19A	89	60.67	0.01058	0.00342	7486	2423	82	44
LmiSat20A	15	53.33	0.01032	0.02015	43324	84621	12.71	14.15
LmiSat21A	38	50.00	0.01013	0.00194	16790	3222	85	91
LmiSat22A	17	58.82	0.01000	0.00923	37056	34220	10.81	10.28
LmiSat23A	223	61.43	0.00927	0.01061	2618	2998	42	73
LmiSat24A	266	56.39	0.00895	0.00656	2120	1553	2.06	5.14
LmiSat25A	219	39.73	0.00558	0.00675	1605	1943	48	35
LmiSat25B	220	37.27	0.00276	0.00374	791	1070	2.79	6.11
LmiSat26A	240	66.52	0.00544	0.00224	1434	591	53	52
LmiSat26B	240	65.83	0.00359	0.00108	941	284	3.78	4.07
LmiSat27A	57	47.37	0.00790	0.01029	8729	11377	99	66
LmiSat28A	263	57.41	0.00532	0.00962	1275	2303	1.23	1.62
LmiSat28B	242	55.79	0.00236	0.00429	614	1117	94	57
LmiSat29A	68	58.82	0.00719	0.00193	6659	1786	9.36	14.48
LmiSat30A	138	40.58	0.00680	0.00550	3102	2511	74	03
LmiSat31A	8	50.00	0.00427	0.00001	33647	79	3.25	40.01
LmiSat31B	10	50.00	0.00161	0.00002	10122	116	76	2 57
LmiSat31C	11	45.45	0.00080	–	4567	–	3.55	–
LmiSat32A	261	51.72	0.00631	0.00565	1523	1363	98	18
LmiSat33A	21	47.62	0.00627	0.00394	18820	11817	7.77	8.35
LmiSat34A	299	61.87	0.00622	0.00475	1312	1001	81	39
LmiSat35A	228	55.70	0.00597	0.00529	1649	1463	2.43	4.64
LmiSat36A	15	60.00	0.00367	0.00603	15423	25322	1 84	1 39
LmiSat36B	15	60.00	0.00218	0.00331	9168	13920	16.94	14.65
LmiSat37A	238	65.97	0.00451	0.00328	1193	867	1 12	1 85
LmiSat38A	42	64.29	0.00511	0.00463	7668	6949	14.56	14.94
LmiSat39A	53	32.08	0.00503	0.00130	5984	1551	79	17
LmiSat40A	148	67.57	0.00459	0.00229	1954	975	2.35	3.05
LmiSat41A	180	61.67	0.00455	0.00579	1592	2026	38	14
LmiSat42A	127	51.18	0.00447	0.00123	2218	610	2.02	4.60
LmiSat43A	231	53.68	0.00440	0.00003	1199	8	68	57
LmiSat44A	17	29.41	0.00428	0.00050	15869	1843	11.45	11.30
LmiSat45A	274	54.01	0.00420	0.00657	966	1510	20	22
LmiSat46A	353	59.77	0.00407	0.00710	727	1267	15.49	11.38
LmiSat47A	41	41.46	0.00369	0.00580	5675	8909	1 46	1 22
LmiSat48A	220	58.18	0.00366	0.00112	1048	322	3.80	7.74
LmiSat49A	47	42.55	0.00362	0.01129	4859	15133	24	70

			Abundance		Repeats		Divergence	
Variant	Length	A+T	SL	NL	SL	NL	SL	NL
LmiSat50A	16	56.25	0.00311	0.01631	12239	64229	8.27	8.23
LmiSat50B	16	43.75	0.00020	0.00059	780	2332	8.90	8.52
LmiSat51A	241	63.90	0.00294	0.00583	769	1524	7.32	3.97
LmiSat52A	143	51.75	0.00257	0.00758	1134	3340	22.15	14.01
LmiSat53A	47	40.43	0.00248	0.01904	3328	25520	3.16	5.20
LmiSat54A	272	56.25	0.00244	0.00512	565	1187	4.55	4.15
LmiSat55A	90	35.56	0.00164	0.00740	1147	5182	15.62	8.57
LmiSat56A	19	52.63	0.00047	0.00153	1558	5063	4.86	4.25
LmiSat56B	19	47.37	0.00029	0.00305	970	10103	5.31	4.74
LmiSat56C	22	45.45	0.00007	0.00108	202	3102	5.53	4.11
LmiSat56D	21	52.38	–	0.00102	–	3054	–	3.34
LmiSat57A	230	63.04	0.00052	0.00470	142	1286	18.21	3.40
LmiSat58A	86	41.86	0.00008	0.01273	56	9327	5.99	3.12
LmiSat59A	16	43.75	0.00004	0.00101	175	3978	18.23	15.88
LmiSat59B	16	31.25	–	0.00337	–	13254	–	14.39
LmiSat59C	16	43.75	–	0.00054	–	2136	–	13.02
LmiSat60A	255	52.94	0.00004	0.00527	10	1302	1.03	0.99
LmiSat61A	63	42.86	0.00002	0.00617	21	6171	14.99	4.60
LmiSat62A	23	43.48	–	0.00450	–	12338	–	4.57

Table S2: Length (bp), A+T content (%), abundance (% of the genome), number of repeats calculated as "[abundance x genome size (6.3 Gb)]/repeat length", and divergence (%) for all satDNA variants found in the gDNA libraries analyzed from Southern (SL) and Northern (NL) lineages.

	L1	L2	X	M3	M4	M5	M6	M7	M8	S9	S10	S11	Total
Short satDNAs	0	1	0	4	1	0	0	2	0	6	0	1	15
Long satDNAs	0	7	1	0	1	1	0	2	4	1	0	1	18
Total	0	8	1	4	2	1	0	4	4	7	0	2	33

Table S3: Number of chromosome-specific short and long satDNA families. Note that L2 and S9 chromosomes showed the highest number of exclusive satDNAs.

		chromosome no.												
satDNA	Length (nt)	1	2	X	3	4	5	6	7	8	9	10	11	EI
LmiSat04-18	18										id		i	0.50
LmiSat10-9	9										p		p	1
LmiSat31-8	8										p	p		1
LmiSat56-19	19							p			i			0
**Short**														**0.63**
LmiSat01-193	193	p	p	p	p	p	p	p	p	p	p	p	p	1
LmiSat02-176	176			p			p		p		p	p	p	1
LmiSat03-195	195	p			p									1
LmiSat05-400	400										id		p	0
LmiSat06-185	185		p		p					p	p		p	1
LmiSat14-216	216				i,i								i	1
LmiSat23-223	223		d					d			i			0.33
LmiSat37-238	238	i											p	0
LmiSat45-274	274	p,i		p	p									1
LmiSat54-272	272			p	i			i	p	d				0.2
**Long**														**0.65**

Table S4: Calculation of the equilocality index (EI) for short and long satDNAs. Only 4 short and 10 long satDNAs showed loci in more than one chromosome pair, and this allows testing the equilocality of satDNA distribution. Among the four short satDNAs, LmiSat56-19 showed a proximal cluster on the M6 chromosome and an interstitial one on S9, thus showing absence of equilocal distribution (equilocality index: EI= 0). By contrast, LmiSat10-9 and LmiSat31-8 showed one proximal cluster on two different chromosomes thus displaying full equilocal distribution (EI= 1). Finally, LmiSat04-18 showed interstitial and distal locations on S9 and interstitial on S11. Out of the two possible pairwise comparisons (i.e. S9i with S11i, and S9d with S11i) only the first one was equilocal, so that EI= 0.5 in this case. The average EI for the four short satDNAs was thus 0.63. In the case of long satDNAs, six of them showed full equilocality (LmiSat01-193, LmiSat02-176, LmiSat03-195, LmiSat06-185, LmiSat14-216 and LmiSat45-274), two showed absence of equilocality (LmiSat05-400 and LmiSat37-238) and two showed intermediate situations: LmiSat23-223 was distally located on two chromosome pairs and interstitially on another pair, so that only one out the three possible pairwise comparisons was equilocal (EI= 1/3). On the other hand, LmiSat54-272 was proximally located on two chromosome pairs, interstitially on two others and distally on another pair. Therefore, only two out of the ten possible pairwise comparisons were equilocal (EI= 0.2). On average, the ten long satDNAs showed 0.65 equilocality index, which is very similar to that calculated for short satDNAs.

Family	Sequence	Length (nt)	Occurences	Number per genome
LmiSat31A-8	CTGTGACT	8	31518697	198568
LmiSat31B-10	CTGTGACTCT	10	1769538	11148
LmiSat31C-11	CTGTGACGACT	11	375521	2366
LmiSat50A-16	CTAGTGTAACTCTGTG	16	549	3
LmiSat50B-16	CGAGTGTAACTCTGCG	16	211	1
LmiSat59A-16	CTGTGATGACTCTGGG	16	244	2
LmiSat59B-16	CTGTGACCACTCCGGG	16	155	1
LmiSat59C-16	TTGTGACCACTCCGTG	16	406	3
LmiSat31A-8 dimer	CTGTGACTCTGTGACT	16	471	3
LmiSat31B-8 dimer	CTGTGACTCTCTGTGACTCT	20	0	0
LmiSat31C-8 dimer	CTGTGACGACTCTGTGACGACT	22	0	0
LmiSat50A-16 dimer	CTAGTGTAACTCTGTGCTAGTGTAACTCTGTG	32	0	0
LmiSat50B-16 dimer	CGAGTGTAACTCTGCGCGAGTGTAACTCTGCG	32	0	0
LmiSat59A-16 dimer	CTGTGATGACTCTGGGCTGTGATGACTCTGGG	32	0	0
LmiSat59B-16 dimer	CTGTGACCACTCCGGGCTGTGACCACTCCGGG	32	0	0
LmiSat59C-16 dimer	TTGTGACCACTCCGTGTTGTGACCACTCCGTG	32	0	0

Table S5: Extremely short satDNAs can arise by chance in the huge genome of *L. migratoria*. This table shows the number of occurrences found for satDNA variants, belonging to Superfamily 5, in 159 genomes randomly generated *in silico*, and searched for as monomers and dimers. Note that the LmiSat31A-8 monomer showed very high likelihood of arising by chance, and it even appeared three times as a dimer. For longer variants of this satDNA (B and C), however, dimers were not observed in the random genomes. The two other satDNAs (LmiSat50-16 and LmiSat59-16) were barely found as monomers but not as dimers.

Family	RepBase
LmiSat02-176	Helitron-N14_LMi
LmiSat06-185	CCRP1
LmiSat15-190	Gypsy-53_LMi-I
LmiSat19-89	DNA-5_LMi
LmiSat29-68	hAT-30_LMi
LmiSat34-299	Polinton-1_LMi
LmiSat55-90	hAT-13_LMi

Table S6: Homology of *L. migratoria* satDNAs with other Orthoptera sequences in Repbase. All the matches are transposons described in *L. migratoria*, except the CCRP1 satDNA from *Caledia captiva*.

SF	satDNA Family	Length	A+T	V	Abundance	Divergence	Heckmann et al.[16]
	LelSat01-43	43	51.16	1	2.48845	7.18	CL21Contig28_X, CL9Contig39_X
2	LelSat02-4	4	75.00	1	1.50044	2.96	CL72Contig1
	LelSat03-150	150	62.00	1	1.18933	8.54	CL7Contig1
1	LelSat04-228	228	66.67	1	1.01380	6.36	–
	LelSat05-56	56	66.07	1	0.64527	9.43	CL11Contig68_X
1	LelSat06-359	359	64.62	2	0.59017	5.88	CL4Contig63
	LelSat07-42	42	47.62	1	0.52823	7.83	CL27Contig80_X
	LelSat08-41	41	41.46	1	0.44139	9.59	CL89Contig6
1	LelSat09-189	189	64.02	1	0.36094	6.14	CL4Contig269_X
	LelSat10-6	6	66.67	1	0.35370	8.58	CL36Contig19_X
	LelSat11-161	161	62.11	3	0.34232	4.72	CL17Contig4
	LelSat12-609	609	72.58	1	0.33025	8.13	CL18Contig96
	LelSat13-6	6	83.33	1	0.31573	3.32	CL25Contig1_X
	LelSat14-68	68	45.59	1	0.27338	5.72	–
	LelSat15-51	51	56.86	1	0.22059	8.20	CL22Contig21
4	LelSat16-179	179	49.72	2	0.20617	7.22	–
	LelSat17-137	137	61.31	1	0.20455	8.94	CL38Contig36
	LelSat18-57	57	71.93	2	0.19652	4.87	CL23Contig24_X
3	LelSat19-189	189	67.72	1	0.12367	4.35	CL43Contig13
	LelSat20-173	173	82.66	2	0.10765	7.17	CL16Contig7
	LelSat21-392	392	70.66	1	0.10619	3.71	CL28Contig19
	LelSat22-374	374	80.48	1	0.10563	7.59	–
	LelSat23-195	195	81.54	2	0.10466	4.20	CL16Contig6
5	LelSat24-344	344	69.48	1	0.10101	2.97	–
	LelSat25-726	726	68.87	1	0.08733	5.56	CL28Contig14
3	LelSat26-141	141	64.54	1	0.08230	7.47	–
5	LelSat27-203	203	79.31	1	0.07539	3.22	–
	LelSat28-89	89	69.66	1	0.07401	6.23	CL63Contig1
	LelSat29-66	66	39.39	1	0.06857	8.32	–
	LelSat30-42	42	61.90	1	0.05742	8.28	–
	LelSat31-45	45	46.67	1	0.04366	9.63	–
	LelSat32-180	180	70.00	1	0.04090	14.07	CL99Contig6
	LelSat33-82	82	79.27	1	0.03252	6.19	–
	LelSat34-33	33	81.82	1	0.03184	6.62	CL109Contig15

SF	satDNA Family	Length	A+T	V	Abundance	Divergence	Heckmann et al.[16]
	LelSat35-45	45	64.44	1	0.03128	7.78	–
	LelSat36-37	37	48.65	2	0.03091	14.14	–
	LelSat37-42	42	57.14	1	0.03069	5.04	–
	LelSat38-177	177	75.71	1	0.03050	6.38	–
	LelSat39-43	43	65.12	1	0.03001	5.15	–
	LelSat40-99	99	54.55	1	0.02503	13.17	–
	LelSat41-541	541	63.22	1	0.02077	5.96	–
3	LelSat42-89	89	60.67	1	0.01730	4.02	–
	LelSat43-108	108	58.33	1	0.01696	5.47	–
	LelSat44-6	6	66.67	2	0.01619	7.92	–
	LelSat45-64	64	62.50	1	0.01439	5.58	–
3	LelSat46-37	37	62.16	1	0.01357	13.70	–
2	LelSat47-6	6	83.33	2	0.01323	4.59	–
3	LelSat48-228	228	65.35	1	0.01267	11.63	–
	LelSat49-218	218	71.10	1	0.01218	3.94	–
	LelSat50-58	58	53.45	1	0.01180	8.02	–
4	LelSat51-213	213	50.23	1	0.01067	11.27	–
	LelSat52-42	42	69.05	1	0.01032	6.86	–
	LelSat53-107	107	48.60	1	0.01011	10.01	–
	LelSat54-113	113	74.34	2	0.01000	4.31	–
	LelSat55-7-tel	7	57.14	1	0.00913	7.52	–
3	LelSat56-76	76	65.79	1	0.00905	15.89	–
	LelSat57-137	137	57.66	1	0.00683	12.68	–
	LelSat58-82	82	29.27	1	0.00618	11.93	–
	LelSat59-107	107	80.37	1	0.00610	9.67	–
	LelSat60-18	18	72.22	2	0.00574	9.25	–
	LelSat61-21	21	61.90	1	0.00562	7.67	–
	LelSat62-66	66	56.06	1	0.00521	3.68	–
3	LelSat63-129	129	60.47	1	0.00514	8.23	–
	LelSat64-108	108	67.59	1	0.00502	11.29	–
	LelSat65-196	196	51.02	1	0.00458	6.93	–
	LelSat66-141	141	48.23	1	0.00435	5.93	–
	LelSat67-261	261	63.98	1	0.00412	10.17	–
	LelSat68-6	6	66.67	2	0.00401	6.26	–
	LelSat69-30	30	36.67	1	0.00281	12.73	–
	LelSat70-129	129	63.57	1	0.00259	7.13	–

SF	satDNA Family	Length	A+T	V	Abundance	Divergence	Heckmann et al.[16]
	LelSat71-232	232	69.40	1	0.00254	10.70	–
	LelSat72-39	39	41.03	1	0.00254	8.45	–
	LelSat73-62	62	74.19	1	0.00246	9.48	–
	LelSat74-186	186	61.83	2	0.00244	6.91	–
	LelSat75-141	141	63.12	2	0.00240	7.65	–
	LelSat76-60	60	31.67	1	0.00240	9.28	–
	LelSat77-55	55	49.09	1	0.00238	5.87	–
5	LelSat78-77	77	81.82	1	0.00220	9.53	–
	LelSat79-56	56	66.07	1	0.00182	4.57	–
	LelSat80-309	309	54.37	1	0.00156	2.95	–
	LelSat81-23	23	43.48	1	0.00149	15.38	–
	LelSat82-30	30	33.33	1	0.00137	9.16	–
	LelSat83-166	166	60.24	1	0.00109	8.62	–
	LelSat84-82	82	71.95	1	0.00035	5.93	–
	LelSat85-115	115	50.43	1	0.00035	13.42	–
	Total	–	–	100	12.92641	–	–

Table S7: Characterization of the *Luzula elegans* satellitome. Length (nt), A+T content (%), number of variants (V), abundance (% of the genome), divergence (%) and equivalency with satDNA analyzed by Heckmann et al.[16].

satDNA	Forward	Reverse
LmiSat01-193	ACGAAAATCATCTGCTCCTTGA	TTGTTACCATGGGCCAGGGA
LmiSat02-176	GCCATCTTCCTGCACCTCCTCCT	CGTGTCTCCTGTAGCGTGAGTGG
LmiSat03-195	GCACTCCAGCGTCCATTCTGTCG	GCGAGCTGCACTGGCGACTA
LmiSat04-18	AAACCACTGTCTTGTGCG	CACAAGACAGTGGTTTCG
LmiSat05-400	TCCCATCGTTCCAAATTCACCC	CGCCAGGAGGCACGAAAG
LmiSat06-185	AGCCGTCGCCACATGACACT	CATTTCGGAGCGAGGCCGGA
LmiSat07-5	GGTTAGGTTAGGTTAGGTTA	TAACCTAACCTAACCTAACC
LmiSat08-168	ACCCCACTTTCAAGAAATTTAATTCT	GTGCTGCCAGTGGGTGCA
LmiSat09-181	TTCTCAACATTCCGGTCGCC	CGTTATCTGACCTTCCTTTAGTCG
LmiSat10-9	CGTCAATGTCGTCAATGTCG	GACATTGACGACATTGACGA
LmiSat11-37	CTCTCTCTCTCCGAAAATTTATATTC	AGAGAGAGAGAGAGAGAGAGA
LmiSat12-273	AGCGATGTGAAGCAGATGGC	GAAAACACCAGTCACAGCCG
LmiSat13-259	CCTTGCCACAACCTACCGTT	GCGTACCAATAGGCTGCTCT
LmiSat14-216	AGAAAATGCAGCCGAGAGCT	GGTGTCTCCACGTAATCGGC
LmiSat15-190	TGCCAATAGAAGAGCATGCAG	GCAGGGTCTGGAAATGTTCTGA
LmiSat16-278	TAGTTGCCCATTTACGGGCA	CCTCCTCCCCTTACACCCTG
LmiSat17-75	TGGTAAGAAGGGTCAAGTACAGGT	CACTACATTCTCAATAGTGAGCCT
LmiSat18-210	GAGCTGCTGGAGGCAACG	TCGTACAGCCCCTCCCTCTAT
LmiSat19-89	AGGAGAAGTAATTAAGCAATGCA	CCTACTACGTGTTGTCGAGC
LmiSat20-15	GGCAAGTATGCTTGTGGC	GCCACAAGCATACTTGCC
LmiSat21-38	GCCTCACTGCTGAGCTTTTGTATACG	CAGTGAGGCAACGCCAGGTAAC
LmiSat22-17	GGGAAAAACGCAGATATGGG	CCCATATCTGCGTTTTTCCC
LmiSat23-223	TAGTCTGCAGTGGCCAGGTG	TGCCTCTGCCCTCACTAGTC
LmiSat24-266	CTGGCACCGTCCACCCACC	CTCCAGAAGCGGCGGCTGG
LmiSat25-219	TGCGTCCTCGAGTCATCCTCG	GCACAAGCTAATACGCCGCCA
LmiSat26-240	CGTTCAGTGGACATTCGTAA	ACGATGCCTGGGCTACGAC
LmiSat27-57	TGGCGGGCCGTGGCATCC	ACCTGACCGCCTCCAAACTCCA
LmiSat28-263	CGCTTGAGTGCCGTTCTTCAGGT	CGCCCGAAACTAGCATGTATATGTGT
LmiSat29-68	GTGGCTGCGGCTAGACTGGC	CACGGCATCAGCGCAGCG
LmiSat30-138	TCACAGAGTCACAGAGTCACAGAG	CTCTGTGACTCTGTGACTCTGTGA
LmiSat31-8	CTCGCCCAACGTAGACTACAGC	GAGGAGCCGCACAGAGCGG
LmiSat32-261	CCGTACACGCTTAGCGAATCTCCG	CGGAGATTCGCTAAGCGTGTACGG
LmiSat33-21	GGTGTCTCCAGCTGAACAGATG	AGATTCATCATACTTGATTTCAAACA
LmiSat34-299	TCCACCCTTTGTTTCATTGGAGT	GAAATAAAAGCAACAACTAAAAACAAC
LmiSat35-228	CCAACATACTATGAGCCAACATACTA	TAGTATGTTGGCTCATAGTATGTTGG
LmiSat36-15	ATTACGTCTATAAGATTACGAAA	ACGGCGCCAGAGATAATTTCG
LmiSat37-238	GCACTGTCATCCGATAATTAGGT	GCACTAATTCGAACATCTAATTTTTCT
LmiSat38-42	CGTCTATAAGATTACGAAATTATCTCT	TTACGGCGCCAGAGATAA
LmiSat39-53	TGGGAGAGGCGTGTGGAGGC	CACTGCTCGGCGACTGGCC
LmiSat40-148	ACCGTCACCACCAGTAGAGG	GGAGCCATTTCTGAAACAACCC
LmiSat41-180	ACTGAAAATAGGAAAATCCAGAGCCTC	AGTGTTTCAGGGATGTGTGTACTACA
LmiSat42-127	GCAGCATCGGTCTTCCTCTCTTTCG	GCACTCACCTCGGAAACTTCCACA
LmiSat43-231	CCAATGCAGACAACTGAAGGCAAC	TGGTATAGACGCTTTCCGGCGT
LmiSat44-17	CAGCCCTTCTGGACGGCC	CCGTCCAGAAGGGCTGGC
LmiSat45-274	ACGGAGGAGGTCATGTTTGCTGG	ACAAACGGCACTGAGCTTCCGA
LmiSat46-353	CAAATGGTACGTCACACATAAAATGGT	TTTTAACGTCATGCGCCTTCAC
LmiSat47-41	GACAGCAGTGGAATGCGCAGC	TCTCCACTCCTCCACAAACGC
LmiSat48-220	AGCACCACAGCGCTACATTT	GCTGCAAAACACAGTGGTCTG
LmiSat49-47	CCCCCTCTCCTTCTATACCACACG	GGGAAGCGGAGAAGGCAGGA
LmiSat50-16	AGCACAGAGTTACACTAGCAC	GTGCTAGTGTAACTCTGTGCT
LmiSat51-241	GCCCAGAGGAGCGTCAAGTGG	ACTGCGACGTTGGACCTGGA
LmiSat52-143	TCTGAGGCTGAACAGGCTGCC	ACACCGTCAAGCAAATGCAGCA
LmiSat53-47	CTCGCTGCTGAACAGAGCCA	AGCAACTTCACCAGCAGCGC
LmiSat54-272	TACAGGAGGCCGGCGGCAG	CAGCGCGCACCTCCCTCCTC
LmiSat55-90	GGCACACACAGTGGCGAGGG	GCCGCCGTGTTCAGCAGAGA
LmiSat56-19	CTCCTGTATACCTGCACTG	CAGTGCAGGTATACAGGAG
LmiSat57-230	TGCTACTCCACATAAAGATCGTGAG	TCTTCTTATGTTACTGTTCTGAGGCA
LmiSat58-86	TGCTGCCTTACAGCGTTGCG	AGGAGGGAAAGGGGCGTGAAC
LmiSat59-16	TCACAGCCCGGAGTGGTCACA	TGTGACCACTCCGGGCTGTGA
LmiSat60-255	GCAGCAGGATGAGCAAGGACGG	GCGGTGAAGAACTCTCCCCTGG
LmiSat61-63	GGGACGTGTGCTGTTATCAGTGGG	CCCTACCTGCAGCGTAACCAAGC
LmiSat62-23	AGGCAGCGAGGGCTCTGTTC	AGCCCTCGCTGCCTTATGAA

Table S8: Primers designed in this study to amplify each satDNA family.

Sequence	Length (nt)	Occurences	Number per genome
ATACAAGC	8	33632104	211882
ATACAAGCT	9	9622569	60622
ATACAAGCTT	10	3159365	19904
ATACAAGCTTA	11	813956	5128
ATACAAGCTTAA	12	266972	1682
ATACAAGCTTAAC	13	58277	367
ATACAAGCTTAACC	14	12023	76
ATACAAGCTTAACCC	15	2465	16
ATACAAGCTTAACCCG	16	451	3
ATACAAGCTTAACCCGT	17	157	1
ATACAAGCTTAACCCGTC	18	32	0
ATACAAGCTTAACCCGTCA	19	8	0
ATACAAGCTTAACCCGTCAT	20	4	0
ATACAAGCTTAACCCGTCATG	21	1	0
ATACAAGCTTAACCCGTCATGG	22	0	0
ATACAAGCTTAACCCGTCATGGT	23	0	0
ATACAAGCTTAACCCGTCATGGTA	24	0	0

Table S9: Frequency of repeats of different lengths observed in the simulated *L. migratoria* genomes. Note that sequences of 15 bp or less are present 16 or more times, indicating that many copies can independently arise by chance. We analyzed ~159 genomes randomly generated *in silico* and searched for a random sequence successively adding a nucleotide, preserving the genomic dinucleotide frequency.

References

1. Wilmore, P. J. & Brown, A. K. Molecular properties of orthopteran DNA. Chromosoma **51**, 337-345 (1975).

2. Ma, C. *et al.* Mitochondrial genomes reveal the global phylogeography and dispersal routes of the migratory locust. Mol. Ecol. **21**, 4344-4358 (2012).

3. Dennis, E. S., Peacock, W. J., White, M. J. D., Appels, R. & Contreras, N. Cytogenetics of the parthenogenetic grasshopper *Warramaba virgo* and its bisexual relatives. VII. Evidence from repeated DNA sequences for a dual origin of *W. virgo*. Chromosoma **82**, 453–469 (1981).

4. John, B., Appels, R. & Contreras, N. Population cytogenetics of *Atractomorpha similis*. II. Molecular characterisation of the distal C-band polymorphisms. Chromosoma **94**, 45–58 (1986).

5. Arnold, M. L., Appels, R. & Shaw, D. D. The heterochromatin of grasshoppers from the *Caledia captiva* species complex. I. Sequence evolution and conservation in a highly repeated DNA family. Mol. Biol. Evol. **3**, 29–43 (1986).

6. Arnold, M. L. & Shaw, D. D. The heterochromatin of grasshoppers from the *Caledia captiva* species complex. Chromosoma **93**, 183-190 (1985).

7. Rafferty, J. A. & Fletcher, H. L. Sequence analysis of a family of highly repeated DNA units in *Stauroderus scalaris* (Orthoptera). Int. J. Genome Res. **1**, 1–16 (1992).

8. Rodríguez Iñigo, E., Fernández-Calvín, B., Capel, J. & García de la Vega, C. Equilocality and heterogeneity of constitutive heterochromatin: *in situ* localization of two families of highly repetitive DNA in *Dociostaurus genei* (Orthoptera). Heredity **76**, 70–76 (1996).

9. Bachmann, L., Venanzetti, F. & Sbordoni, V. Characterization of a species-specific satellite DNA family of *Dolichopoda schiavazzii* (Orthoptera, Rhaphidophoridae) cave crickets. J. Mol. Evol. 39, 274–281 (1994).

10. Bachmann, L., Venanzetti, F. & Sbordoni, V. Tandemly repeated satellite DNA of *Dolichopoda schiavazzii*: A test for models on the evolution of highly repetitive DNA. J. Mol. Evol. 43, 135–144 (1996).

11. López-León, M. D., Vázquez, P., Hewitt, G. M. & Camacho, J. P. M. Cloning and sequence analysis of an extremely homogeneous tandemly repeated DNA in the grasshop-

per *Eyprepocnemis plorans*. Heredity **75**, 370–375 (1995).

12. Yoshimura, A., Nakata, A., Kuro-o, M., Obara, Y. & Ando, Y. Molecular cytogenetic characterization and chromosomal distribution of the satellite DNA in the genome of *Oxya hyla intricata*; (Orthoptera: Catantopidae). Cytogenet. Genome Res. **112**, 160–165 (2006).

13. Yoshimura, A., Nakata, A., Mito, T. & Noji, S. The characteristics of karyotype and telomeric satellite DNA sequences in the cricket, *Gryllus bimaculatus* (Orthoptera, Gryllidae). Cytogenet. Genome Res. **112**, 329–336 (2006).

14. Pita, M. *et al.* *Arcyptera fusca* and *Arcyptera tornosi* repetitive DNA families: whole-comparative genomic hybridization (W-CGH) as a novel approach to the study of satellite DNA libraries. J. Evol. Biol. 21, 352–361 (2008).

15. Camacho, J. P. M. *et al.* A step to the gigantic genome of the desert locust: chromosome sizes and repeated DNAs. Chromosoma **124**, 263-275 (2015).

16. Heckmann, S. *et al.* The holocentric species *Luzula elegans* shows interplay between centromere and large-scale genome organization. Plant J. 73, 555–565 (2013).
